# Structure of sweet potato (*Ipomoea batatas*) diversity in West Africa covaries with a climatic gradient

**DOI:** 10.1371/journal.pone.0177697

**Published:** 2017-05-26

**Authors:** Kodjo Glato, Atsou Aidam, Ndjido Ardo Kane, Diallo Bassirou, Marie Couderc, Leila Zekraoui, Nora Scarcelli, Adeline Barnaud, Yves Vigouroux

**Affiliations:** 1 University of Lomé, Lomé, Togo; 2 Institut Sénégalais de Recherches Agricoles (ISRA), Dakar, Sénégal; 3 Laboratoire Mixte International Adaptation des Plantes et microorganismes aux Stress Environnementaux (LAPSE), Dakar, Sénégal; 4 Institut de Recherche pour le Développement (IRD), Montpellier, France; Università Politecnica delle Marche, ITALY

## Abstract

Sub-Saharan agriculture has been identified as vulnerable to ongoing climate change. Adaptation of agriculture has been suggested as a way to maintain productivity. Better knowledge of intra-specific diversity of varieties is prerequisites for the successful management of such adaptation. Among crops, root and tubers play important roles in food security and economic growth for the most vulnerable populations in Africa. Here, we focus on the sweet potato. The Sweet potato (*Ipomoea batatas*) was domesticated in Central and South America and was later introduced into Africa and is now cultivated throughout tropical Africa. We evaluated its diversity in West Africa by sampling a region extending from the coastal area of Togo to the northern Sahelian region of Senegal that represents a range of climatic conditions. Using 12 microsatellite markers, we evaluated 132 varieties along this gradient. Phenotypic data from field trials conducted in three seasons was also obtained. Genetic diversity in West Africa was found to be 18% lower than in America. Genetic diversity in West Africa is structured into five groups, with some groups found in very specific climatic areas, e.g. under a tropical humid climate, or under a Sahelian climate. We also observed genetic groups that occur in a wider range of climates. The genetic groups were also associated with morphological differentiation, mainly the shape of the leaves and the color of the stem or root. This particular structure of diversity along a climatic gradient with association to phenotypic variability can be used for conservation strategies. If such structure is proved to be associated with specific climatic adaptation, it will also allow developing strategies to adapt agriculture to ongoing climate variation in West Africa.

## Introduction

Sub-Saharan agriculture is identified as being particularly vulnerable to ongoing climate change [1]. This is considered unther the climatic context in which African countries have to increase agricultural production to feed their growing populations. Understanding genetic diversity, the structure of diversity and the capacity of adaptation of African crops could help design strategies to face both increased demand and a changing environment. Tuber and root crops represent a large proportion of the diet in Africa and consequently are very important for such an endeavor [2]. Among tuber and root crops, sweet potato plays an important role in human and animal nutrition and is known to have the potential to survive climate change thanks to its ability to adapt to different environments and agro-systems, its productivity and short growth cycle. In addition, sweet potato is receiving increased attention as a rich source of vitamin A [3, 2]. Sweet potato is a staple food, ranking seventh among food crops in annual production in the world [4] and fifth for its caloric contribution in developing countries after rice, wheat, maize and cassava [5]. Although Africa occupies the second position, annual production (19%) remains far below that in Asia, which alone produces 75% of the world annual production [6]. Cultivation of the sweet potato is still low in West Africa, and lacks an inventory of diversity and a conservation plan, as well as a study of its sensitivity to pests and diseases [7].

Local landraces of sweet potato need to be collected, characterized and conserved to improve African production. The sweet potato originated in tropical America [8, 9] and was dispersed around the world principally by human mediated migration; and is now cultivated in most tropical regions [10]. It was introduced to Africa by the Portuguese in the 16th century [11, 12]. Probably first introduced as a tuber in Tanzania [10], the plant then dispersed from East to West Africa [10]. Sub-Saharan Africa is considered to be a secondary center of diversity [13]. However, its genetic characterization is still partial.

Among the tools used for assessing the diversity of African sweet potato, agro-morphological analysis was conducted in Tanzania [14], Kenya [15] and Burkina-Faso [16]. Unfortunately, morphological characteristics can vary because of trait plasticity [17] and it has been shown that the environment can affect the phenotypic traits [18]. Consequently, comparisons between studies of morphological diversity are difficult. Molecular markers make it possible to conduct such comparisons across countries and continents. Analysis of sweet potato diversity using SSRs markers was recently initiated [9, 19, 20].

The questions we addressed in this study are: i) how is sweet potato structured along the environmental gradient from the Tropics to the Sahel? ii) Are some genetic groups associated with specific phenotypic differences? To understand the genetic structure and diversity of sweet potato in West Africa, we focused on two countries, Togo and Senegal, which cover a range of climates. We also performed a phenotypic evaluation to compare genetic structure and morphological differences between samples. This analysis enabled us to identify specific genetic groups showing phenotypic differences and occurring in different climatic conditions.

## Materials and methods

### Plant collection

A total of 132 accessions were collected: 112 from Togo in 2012, and 20 from Senegal in 2014 (S1 Table). The collection was done with permission of doctors Bassirou Diallo (ISRA) from Senegal and Nenonene Amen Yawo (ESA) from Togo. Samples were collected with farmers consent. The aim of the collection was to maximize the variability observed in each village [21]. A mean of 3.56 samples was collected per village. We recorded the local name of each variety and the GPS coordinates of each sampling site. For each accession, a stem was sampled and kept isolated for further genetic and phenotypic experiments. In Senegal, we collected accessions in four villages in the region where most sweet potato is cultivated (region of Saint-Louis). We used the same sampling protocol as in Togo, except that young leaves were collected instead of stems. The GPS coordinates of each sampling site were recorded. A mean of 5 samples were collected per village

### Phenotyping

Field trials were conducted only on Togo accessions in the experimental Agronomic station of University of Lomé (altitude 27 masl. 06°10’27.2” E 001°12’ 39.6”N). The field studies did not involve endangered or protected species. The soil in the field is classified as ferralitic. The climate is characterized by two rainy seasons, leading to two periods of sweet potato cultivation (April-July and September-December). From one to three nodes of each of the 112 accessions were planted (236 cuttings with an average 2.10 per accession). The trial was performed on a 300 m^2^ plot (30 m × 10 m). The space between plants and between rows was 1 m. The experiment was repeated three times, once in 2012 and twice in 2013. The stems from the 2012 field experiment were harvested for use in the 2013 field experiment. The field was weeded manually with a hoe and watered if necessary. The morphological characteristics of the leaf and stem were recorded 90 days after planting.

A total of 21 morphological descriptors [22], were used as agro-morphological characteristics of these three organs: leaf, stem, and root. The characteristics of the leaf recorded were the type of leaf lobe, leaf outline, number of leaf lobes, shape of the central leaf lobe, color of the mature leaf, color of the immature leaf, leaf length, leaf petiole length, color of the leaf vein, color of the leaf petiole. The characteristics of the stem recorded were vine internode length, diameter between nodes, color, port and pubescence. As roots are the idle part of the plant, we collected root characteristics at maturity. Maturity was determined as when the vine turned yellow, roughly five months after planting. The root traits recorded were root shape, main color of the skin, secondary color of the skin, main color of the flesh, secondary color of the flesh, distribution of the secondary color of the flesh.

### DNA extraction and SSR genotyping

We genotyped all 132 individuals sweet potato plants from Togo and Senegal (S1 Table). Leaves were collected from the Togo samples during the 2012 field experiment. DNA was extracted from dried young leaves (10 to 25 mg) following the method of Doyle and Doyle [23] adding 2% (w/v) of cetyl-tri-methyl-ammonium bromide (CTAB) and 2% (w/v) of sodium sulfite. The pellet was left to dry at room temperature and resuspended in 100 μl of 1x Tris EDTA buffer (TE). The concentrations were assessed using NanoDrop 2000/2000C. DNA was checked on 0.8% agarose gel. The DNA solution was stored at -20°C until genotyping.

Twelve microsatellite (SSRs) previously designed for sweet potato [24] were used (S2 Table). The amplification conditions were slightly modified and the fluorochromes for each marker (new conditions listed in S2 Table). All loci were independently amplified using the multiplex PCR kit (Qiagen) in a final volume of 10 μl; 5 μl master mix (1x); 2 μl Q solution (1x); 2 μl DNA; 0.2 μl R primer; 0.2 μl F primer and 0.6 μl Rnase free solution. The PCR amplification program (S2 Table) was from Roullier *et al*.[24] with slight modifications. The PCR was performed on a Techne Gradient TC Plus Thermocycler using the following program: 15 min at 95°C, 35 cycles of 30 s at 94°C, 90 s at hybridization temperature varying from 58°C to 60°C (S2 Table), 60 s at 72°C and 30 min at 60°C. Amplifications were performed on 96-well plates with two negative water controls and four positive DNA controls. The amplification product (4 μl) was checked on 2% agarose gel. SSRs were diluted and pooled for migration on an ABI 3130 XL 16 capillary sequencer. GeneMapper software version 4.1 was used to score alleles. Two different people scored the alleles. Since reading of size might be difficult for hexaploid plants [9], we performed a second independent run and an independent score of alleles. The second dataset was used to assess the reproducibility of the results. Analyses were computed independently on the two datasets, and overall agreement was shown between datasets (see details in the supplementary file). In addition, we used previously genotyped accessions from Tropical America and Oceania [24]. To allow comparison with our sampling number, we randomly sampled 132 accessions both for Tropical America and Oceania. The number of alleles recorded was used to statistically compare diversity in the three areas, West Africa, Tropical America and Oceania.

### Genetic data analyses

#### Diversity

Each SSR allele was binary coded (0 for absence and 1 for presence) following previous approaches used for this hexaploid species [24]. We first compared the diversity observed in Africa with the diversity found in America and Oceania using the same number of samples randomly selected from Roullier *et al*.[25], using both the number of alleles and polymorphic information content (PIC). A Wilcoxon paired test with the number of alleles was used to assess differences in diversity. The PIC of the African accessions was calculated using the binary matrix. For this hexaploid species, the PIC was calculated considering an allele as a locus: PIC = *1 –(P*_*1*_^*2*^ *+ P*_*2*_^*2*^ *+ 2P*_*1*_^*2*^*P*_*2*_^*2*^*)* where *P*_*1*_ and *P*_*2*_ corresponded to the frequency of the presence (1) and absence (0) of an allele. So, PIC = *1- Pi*^*2*^ with Pi the frequency of allele *i*.

The neighbor joining tree method was used with the Euclidean genetic distance among accessions as implemented in PowerMarker 3.25 [26]. The phylogram was visualized using the international tree of life: Itoel Program v2 [27].

#### Genetic structure

Discriminant analysis of principal components (DAPC) implemented in the ADENEGET package [28] using R software 3.1.1 [29] was used to assess the distribution of genetic diversity. For DAPC, we first performed a principal component analysis (PCA) to capture the large number of allele absence/presence (0/1) matrices on the most significant PCA axes. The number of PCA axes retained was based on (1) the a score optimization procedure proposed in the ADEGENET package [28], (2) the cumulative percentage of variance explained by the first selected axes. We then assessed the optimal number of clusters supported by the genetic data using the k-means procedure [28]. A Bayesian information criterion (BIC) was calculated with each model (K = 1 to 20). Membership probabilities for each individual were plotted using the compoplot function of R. We also used STRUCTURE 2.3.4 software [30, 31]. We ran the admixture model with a number of populations from K = 1 to 20 with 15 runs using a 100.000 burn in period and 400.000 Markov Chain Monte Carlo (MCMC) steps.

#### Analysis of the composition of the genetic groups

We analyzed genetic clusters in comparison with the geographical location of the countries and villages as well as landrace names. To assess relationships between agro-morphological traits and genetic clustering, we performed Kruskall-Wallis nonparametric tests. We ran this test with the 21 morphological traits recorded. We considered the genetic group to be associated with morphological differences when the test was significant with a Bonferroni corrected p-value of 0.0024. Wilcoxon paired tests were then performed for each significant trait to assess which specific genetic group differed significantly from the others.

We also assessed the relationship between the clustering and climate variables. To do so, for each accession we downloaded the BIOCLIM data [32] composed of 19 bioclimatic variables and monthly climate data (temperature and precipitation). We performed a principal component analysis to obtain synthetic uncorrelated climatic contrast. The PCA axes were then used to check for a relationship between climate variables and inferred cluster groups using Kruskall-Wallis nonparametric tests.

## Results

### Diversity of sweet potato in West Africa

**Fig 1:**
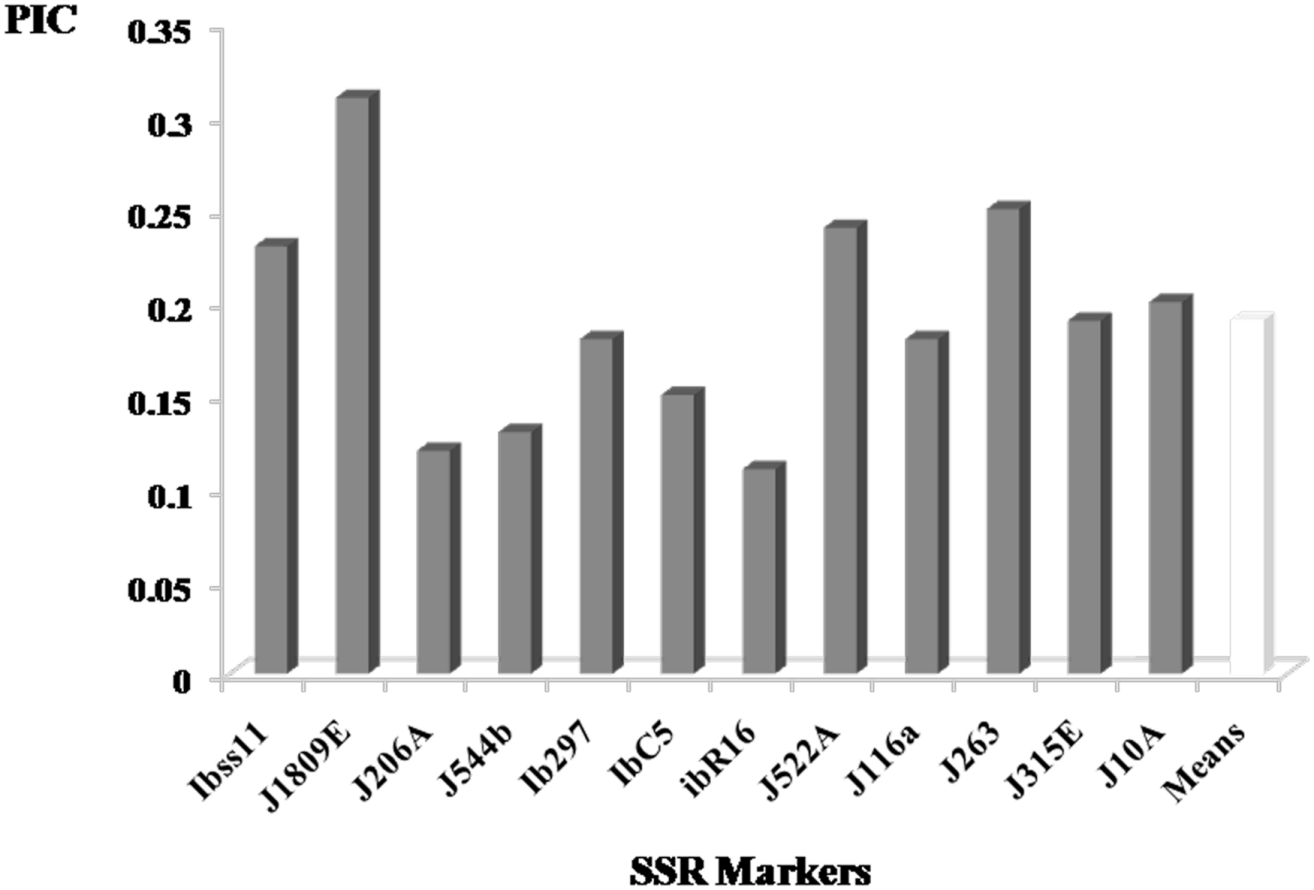
Polymorphic Index Content (PIC) of SSR markers. The values indicate the PIC of the loci used to analyze our 132 African accessions. Each locus value corresponds to the mean PIC of all the alleles of the corresponding markers. The last bar represents the mean PIC of the 12 markers.

**Fig 2:**
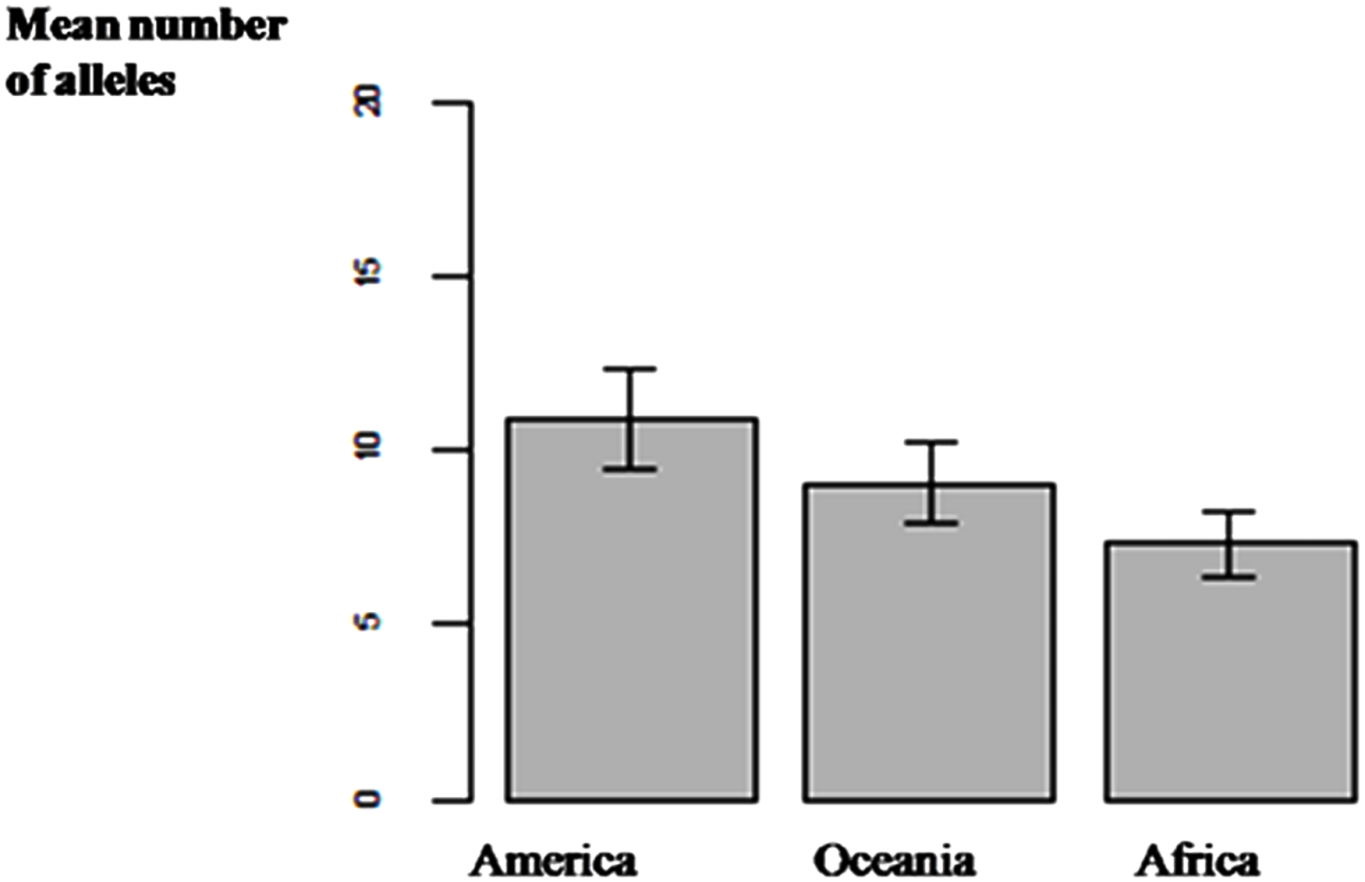
Mean number of alleles in the different regions. America has the highest number of alleles, approximately 11, followed by Oceania with 9 and Africa with 7.

A total of 90 alleles were observed. The number of alleles per locus ranged from 4 to 12 with a mean of 7.5. Locus ib292 had the highest number of alleles and loci J263 and J315 the lowest (S3 Table). The size of the amplified products ranged from 83 bp to 250 bp. Allelic frequency among the accessions ranged from 0.007 to 0.990 and PIC from 0.11 to 0.31 with a mean of 0.19 (Fig 1). The 11 markers used in our study were the same as those used in a previous study [24]. With these 11 SSR markers, we found an average of 7.36 alleles for the West African sample, 9.09 alleles for the Oceanian sample and 10.90 alleles for the American sample (Fig 2). The number of alleles ranged from seven to 22 for Tropical America accessions, four to 17 for Oceanian accessions and four to 12for West African accessions (S4 Table). Allelic frequency ranged from 0.007 to 0.990 in the three regions. The Wilcoxon paired test revealed significant differences in diversity between America and West Africa (p-value = 0.0083) and Oceania (p-value = 0.048). However, no significant difference was found between West Africa and Oceania (S5 Table).

### Structure of sweet potato populations

With the a-score optimization function applied to the dataset, the number of PCA to be retained ranged from two to four. The cumulative variance explained by the 12 first PCs axes was 74.7%, and consequently we retained these 12 axes (S1 Fig). The Bayesian information criterion (BIC) values assessed from two to 12 clusters decreased steadily with increasing K (S2 Fig). We assessed the composition of the group at different K values (3, 4, 5, 6) and K = 5 appeared to be the best number of genetic groups to explain the genetic data on the basis of significant statistical analysis, individual position in the group, and membership probability. Indeed, the DAPC clearly separated individual into groups with K = 3, 4, and 5. But with K = 6, the subdivision of the groups was less clear (S3 Fig). The analysis using STRUCTURE tend also favored five clusters (S4 Fig). In terms of clustering between DAPC and STRUCTURE, the inferences of ancestry between groups were very similar (S6 Table).

**Fig 3:**
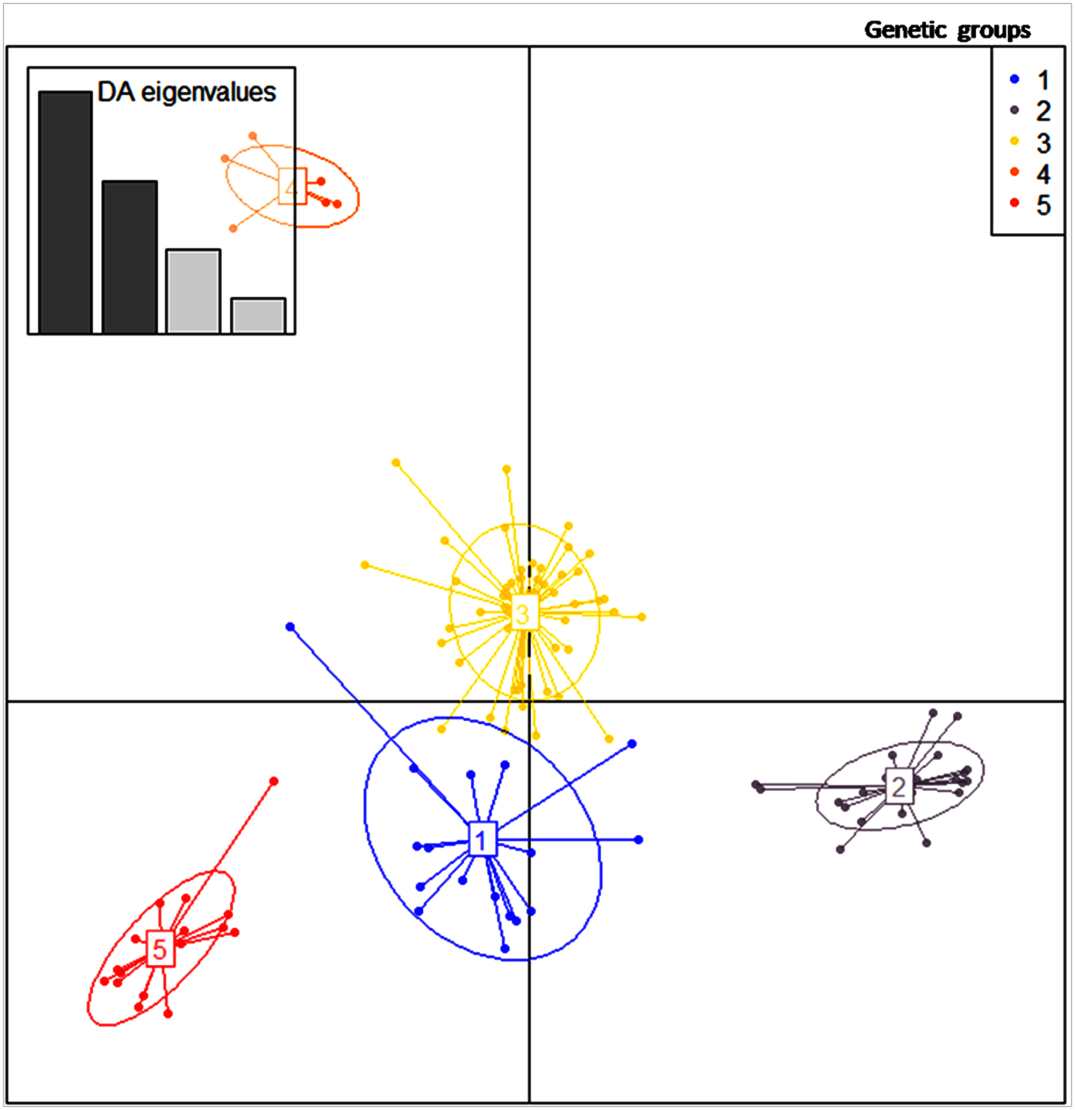
DAPC genetic groups with k = 5. In blue: Tropical-Sahelian accessions group1 with 18 accessions (15 from Togo and 3 from Senegal); in grey: Tropical accessions with 27 accessions (all from Togo); in yellow: Tropical arid accessions with 60 samples (56 from Togo and 4 from Senegal); in pink: Sahelian accessions with 8 accessions (all from Senegal) and in red, Tropical-Sahelian accessions group 2 composed of 19 accessions (14 from Togo and 5 from Senegal).

**Fig 4:**
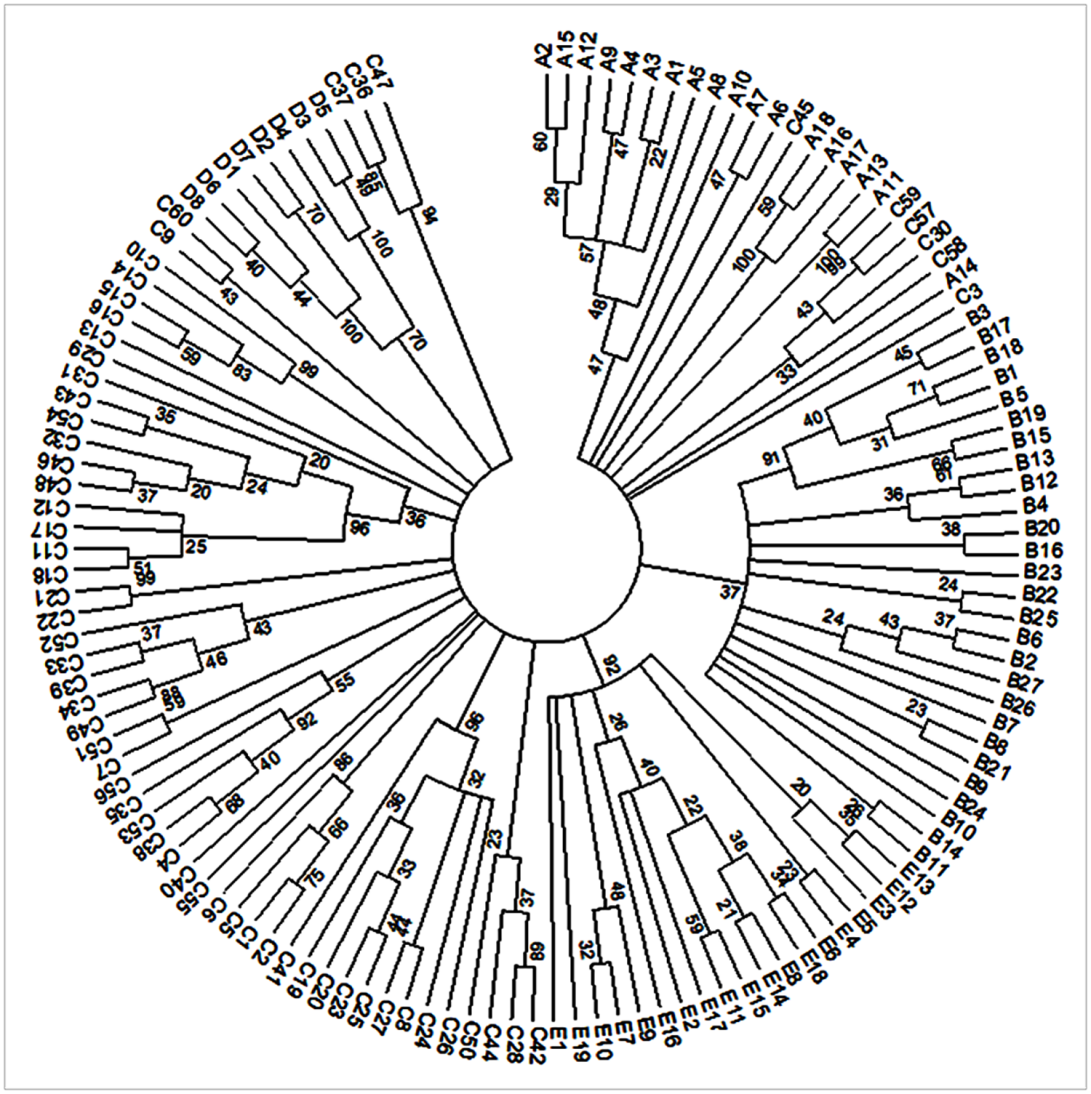
Dendrogram of NJ cluster analysis of 132 sweet potato accessions. A represents Tropical-Sahelian accessions group 1; B Tropical accessions, C Tropical arid accessions, D Sahelian accessions, and E Tropical-Sahelian accessions group 2. The number indicates the number of accessions. The bootstrap value is given on the branch of the tree.

When K = 5 (Fig 3), the first group contained 18 accessions, 15 from Togo and three from Senegal. The second group, called “Tropical accessions”, contained 27 accessions all from Togo. The third group was composed of 60 accessions of which only four were from Senegal. All the accessions from the northern part of Togo were in this group, named “Tropical arid accessions”. Group four contained eight accessions all from Senegal called “Sahelian accessions”. Group five contained 19 accessions including five from Senegal and the rest from Togo. Group one and group five were composed of accessions with no clear specific geographical origin, called “Tropical-Sahelian accessions group 1” and “Tropical-Sahelian accessions group 2” respectively. The membership probabilities of each individual in each group were very high for each accession (S5 Fig, S6 and S7 Tables). The result of the phylogram among accessions was also in agreement with the five genetic groups (Fig 4).

### Local name and genetic groups

A total of 31 different local names were recorded: 18 in Togo and 13 in Senegal. The names given to the accessions by the farmers did not match the genetic groups. Indeed, 76% of accessions with the same local name were in fact attributed to different groups. For example, four accessions called “dankadou” were distributed between the Tropical arid accessions group and Tropical-Saharan accessions group 2. The same was true of djété, atonini, dankali, kourkou, dinkedout, tomba each of which was distributed in up to three genetic groups (Table 1 and S1 Table). The local name and the genetic group agreed in only 25% of the cases.

**Table 1:** Distribution of the genetic group per village.

Villages	Latitude	Longitude	Country	I	II	III	IV	V
GBAVE	N 06°20'08.0"	E 001°01'40.0"	TOGO			1		
AGBODJEKPOE	N 06°39'57.8"	E 001° 09'44.7"	TOGO	1	1	4		
GNAMADJI	N 06° 15'24.4"	E 001°19'05.3"	TOGO	4	3	8		
GBATOKOPE	N 06°14'31.6"	E 001° 32'05.5"	TOGO			1		
BADJA	N 06°23'81.8"	E 000°59'67.6"	TOGO			1		1
ATSANSI DEDZI	N 06°27'26.9"	E 001°32'50.9"	TOGO	1	1	1		
ASSAHOUN	N 06°27'56.0"	E 000°54'16.1"	TOGO		2			
MOM-HAGOU	N 06°29'30.8"	E 001°33'30.9"	TOGO			1		
KAGNIKPEDJI	N 06°38'48.4"	E 001°10'22.4"	TOGO	1				
BAKAKOPE	N 06°39'43.5"	E 000°54'20.5"	TOGO	1				
ADRALAKOPE	N 06°41'22.0''	E 001°08'59.1''	TOGO			2		
GNIGBE	N 06°42'06.6"	E 001°08'14.3"	TOGO		2	8		2
AGAMAHE	N 06°42'40.5"	E 001°10'19.1"	TOGO					2
ADAKAPE	N 06°49'04.1"	E 001°10'52.5"	TOGO					1
KPALIME TSEVIE	N 06°55'22.4"	E 000°38'31.0"	TOGO		1	4		
YOKELE	N 06°56'42.1"	E 000°39'45.5"	TOGO		4			
AKATA	N 07°02'14.6"	E 000°42'20.5"	TOGO	1	3	1		
KPELE-TSIKO	N 07°07'37.6"	E 000°42'13.3"	TOGO		2			1
DANYI-N'DIGBE	N 07°08'30.1"	E 000°40'35.5"	TOGO	2				
DANYI PEYEYEME	N 07°12'42.1"	E 000°41'56.2"	TOGO		1	3		
KPETE	N 07°25'05.6"	E 000°52'57.6"	TOGO		1	1		
TCHEBEBE	N 08°26'16.0"	E 000°59'30.4"	TOGO			2		3
TCHALO	N 08°55'43.9"	E 001°07'03.9"	TOGO		4	3		
BOTCHOLEYO	N 08°56'02.0"	E 001°05'48.8"	TOGO			1		
AMAOUDE	N 09°08'44.2"	E 001°09'24.0"	TOGO			2		
ATCHANGBADE	N 09°28'39.4"	E 001°08'17.2"	TOGO			1		1
DJAMDE	N 09°30'50.6"	E 001°02'40.0"	TOGO	3		2		
TANDJOUARE	N 10°40'15.4"	E 000°12'19.6"	TOGO		1	2		
NAKI-EST	N 10°43'31.3"	E 000°22'37.6"	TOGO			2		1
NABOULPIONGUE	N 10°56'20.8"	E 000°08'01.3"	TOGO			2		1
GABONBONG	N 11°01'22.1"	E 000°04'56.7"	TOGO			3		1
KASSENA	N 08°52’60”	E 001°04’60”	TOGO	1				
KPADAPE	N 06°51’00”	E 000°36’00”	TOGO		1			
GUIDICK	N 16°07'16.4"	W 015°53'52.9"	SENEGAL			2		1
SANEITE	N 16°14'06"	W 015°47'30.2"	SENEGAL	2		2	3	4
MBANN	N 16°17'02.2"	W 015°47'02.9"	SENEGAL	1			1	
DAGANA	N 16°32'27.1"	W 015°30'35.6"	SENEGAL				4	

Each village is shown together with its passport data and country. The color of the genetic groups (I to V) shows if it is present in each village. The number of samples per genetic group in each village is also shown.

### Village richness

**Fig 5:**
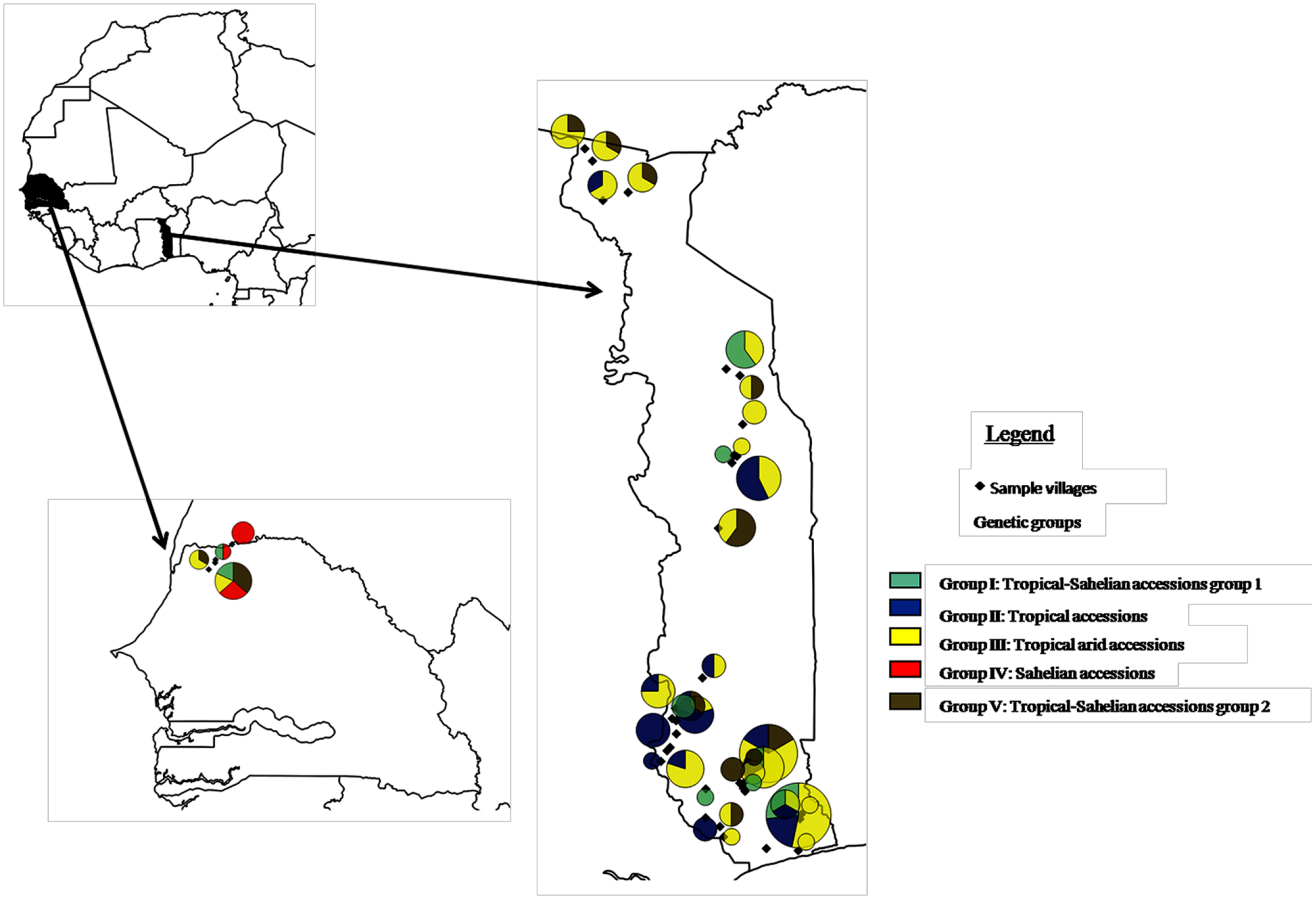
Geographical distribution of the genetic groups. In the villages sampled, each color represents one genetic group. The number of colors in each circle corresponds to the number of genetic groups found in the village concerned. The size of the circle is proportional to the number of samples. The total surface area of each color is also proportional to number of samples in that genetic group.

Different genotypes from different genetic clusters were generally found within the villages. No village had all five genetic groups (Table 1 and S1 Table). Among the 37 villages sampled, only one had four genetic groups, five villages had three genetic groups and 15 villages had two genetic groups. The Tropical arid accessions group was the most widespread across villages and accessions from this group were found in 67% of the villages (Table 1, S1 Table and Fig 5). The least represented group was “Sahelian accessions”, which was found in only 8% of the villages. The other tropical accessions, “Tropical-Sahelian accessions group1” and “Tropical-Sahelian accessions group 2”, were present in 25% of the villages.

### Genetic group and morphological traits

The different genetic groups were also distinguished by the 21 agro-morphological traits (Table 2). For successive values of K from 3 to 5, six agro-morphological traits distinguished a genetic group whatever the K-value: the main color of the skin of the root, leaf outline, the number of lobes, the type of central leaf lobe, the color of the leaf veins and the color of the immature leaf. When K = 3, seven traits were associated. In addition to the six common traits, we found the color of the leaf petiole. For K = 4 and K = 5, nine traits were associated. In each case, in addition to the six common traits, we found the secondary color of the skin, pubescence, type of leaf lobe for K = 4, and petiole color, pubescence, and type of leaf lobe type for K = 5 (Table 2).

**Table 2:** Kruskall-Wallis test between genetic group and agro-morphological traits.

Traits recorded	Codes	K = 3	K = 4	K = 5
Shape of the roots	FGt	0.41	0.53	0.57
Main color of the skin	CPt	2.5*10^−6^	2.9*10^−6^	1.9*10^−6^
Secondary color of the skin	CSt	5.3*10^−3^	3.3*10^−4^	0.01
Main color of the flesh	CPc	0.047	0.01	7.2*10^−3^
Secondary color of the flesh	Csc	0.15	0.02	0.08
Distribution of secondary color of the flesh	Dcsc	0.76	0.86	0.92
Length of vine internodes	Lnoe	0.58	0.62	0.78
Diameter between nodes	Dianoe	0.03	0.08	0.11
Color of the stem	Cpliane	0.01	0.01	0.03
Port	Port	0.48	5.2*10^−3^	0.06
Pubescence	Psomet	0.04	2.07*10^−7^	2.8*10^−7^
Type of leaf lobe	Tylobe	2.9*10^−3^	1.7*10^−3^	6.1*10^−4^
Leaf outline	FGfeuille	3.0*10^−4^	1.3*10^−4^	1.3*10^−4^
Number of leaf lobes	Nlobe	4.8*10^−4^	2.4*10^−4^	6.3*10^−5^
Shape of central leaf lobe	Flobec	1.6*10^−3^	4.3*10^−4^	1.3*10^−4^
Color of mature leaf	Cfm	0.74	0.24	0.5
Color of immature leaf	Cfim	1.1*10^−4^	1.5*10^−4^	1.0*10^−4^
Length of leaf	Lfeui	0.24	0.25	0.25
Length of leaf petiole	Lpétio	0.07	0.03	0.01
Color of leaf vein	CNerv	5.2*10^−4^	1.1*10^−4^	2.3*10^−4^
Color of leaf petiole	Cpétiole	3.5*10^−5^	3.3*10^−3^	1.2*10^−4^
	Total	7	9	9

Twenty-one agro-morphological traits were used. The p-value for each trait is reported. The colored boxes show significant p-values < 0.0024 (Bonferroni corrected p-value) for the traits. The number of group was assumed according to K value: from K = 3 to K = 5.

Wilcoxon-paired test tested with K = 5 genetic groups, revealed specific agro-morphological traits for each genetic group (S8 Table). The trait was considered as specific to a group when the p-value of the test was < 0.05 for paired tests. The result showed that “Tropical-Sahelian accession group 2” was distinguished by the main color of the skin of the root and the color of the immature leaf. “Tropical accession” also had very specific traits: the number of lobes, type of central lobe of the leaf, and pubescence (S8 Table).

### Genetic group and climate

We found a significant relationship between the cluster and climatic variables. The PCA on the climatic variable retrieved the three first axes with of 52.6%, 24.2% and 18.4% variance explained, respectively. The first axis mainly represents the south/north gradient from a tropical humid zone (Danyi-Peyeyeme) to the northern Togo tropical arid zone (Village of Gabonbong), and then to the drier part of Senegal (S6 Fig)

We found an association between the first axis of the PCA and the DAPC group (Kruskal-Wallis, p < 10^−6^). This relationship held even when we only considered the accession from Togo (Kruskal-Wallis, p < 0.002). Only the third axis was significant when the whole sample was tested (Kruskal-Wallis, p < 0.001) but not if we only included Togo (Kruskal-Wallis, p < 0.26).

## Discussion

### Relatively low genetic diversity in West African sweet potato

The SSR markers revealed a high level of polymorphism. These values are comparable to those reported in similar studies on the sweet potato in East Africa [33], with a mean of 6.1 alleles per locus compared to 7.4 found in the present study. Yada *et al*. [18] reported an average of four alleles per locus in 192 accessions from Uganda with 10 SSRs used. Gwandu *et al*. [34] found a mean of four alleles in 57 sweet potato from Tanzania. Based on the mean allele value per locus, which was 7.4 in the present study and those listed elsewhere, we tend to have a higher level of diversity in West Africa. However, one should be cautious, because different sets of SSR markers are used, and type and number of repetitions for the microsatellite motif impact the number of alleles and consequently the diversity assessment [35] Nevertheless, a higher diversity in our study is not unexpected, because we sampled a greater climatic contrast in West Africa and more than one country. Indeed, the climate in Togo is contrasted and diversified: arid in the north and humid in the south. In Senegal (Saint Louis) the climate is Sahelian. In Africa, the main hypothesis is that sweet potato was introduced by explorers from Spain and Portugal in the 16^th^ century in east Africa in Tanzania [11, 12, 10]. Under this scenario, West Africa sweet potato is derived from East African sweet potato. The lower diversity in East Africa than in West Africa suggests that sweet potato was not simply a sub-sample from East Africa but might have been re-introduced into West Africa later on. However, confirming this hypothesis would require not only a similar set of markers but also a very similar sampling strategy. A thorough analysis of sweet potato diversity of the whole Africa remains to be done.

### The genetic groups are partly structured by climate

The genetic groups were closely linked to geographic regions with different climatic conditions. Two clusters were specific to a country: the group called “Sahelian accessions” was linked to Senegal and the group called “Tropical accessions” to Togo. The three other groups were roughly associated with climate: “Tropical-arid accessions”, “Tropical-Saharan accessions group 1” and “Tropical-Saharan accessions group 2”. In the tropical-arid accessions, most of the accessions sampled in Togo were from the northern part of the country, where the climate is arid. The two other clusters “Tropical-Sahelian accessions group 1” and “Tropical-Sahelian accessions group 2” contained accessions either from Togo or Senegal and the climatic conditions were less well defined.

The two groups “Tropical accessions” and “Sahelian accessions” were certainly shaped by ecological conditions, and might reflect specific adaptation to climate and soil. However, genetic bottlenecks created during the introduction and later selection may also have played a role in this genetic differentiation. Environmental and climatic variations have also been shown to be associated with the diversity of cassava in Amazonia [36].

The three other genetic groups: “Tropical arid accessions”, “Tropical-Sahelian accessions group 1” and “Tropical-Saharan accessions group 2” contained accessions from both Togo and Senegal. The relationship between a genetic group and specific geographical features has been previously reported [24]. Some studies done in a smaller region did not always report such results, for example in Uganda [18], in Mozambique [37] and in Puerto Rico [38]. However, we covered a wide range of climates, from the tropical humid area to the Sahel, and we consequently have a good power to show such genetic, geographic and climatic congruence.

### Village richness within genetic groups

We observed that each village cultivated accessions from different genetic groups, but did not differentiate the varieties with specific local names. This can be explained by frequent exchanges between villages in Togo because farmers go to other villages to look for work. They return home with stems they found that had attractive traits or with roots of a plant children like because of its sweetness. Such practices both increase and spread genetic diversity between villages and are particularly important in preserving plant diversity and avoiding genetic erosion. The geographical distribution of allelic diversity appears to be a practical and cost-efficient way to preserve genetic diversity [2].

### Do local names distinguish true genetic groups?

Our study has shown that local names used for sweet potato landraces were not correlated with genetic groups (S1 Table). However, the genetic group was well correlated with very specific morphological traits. The way the plant is named and its importance to the farmers probably explains this observation. As sweet potato is propagated vegetatively, farmers use phenotypic traits [9] or organoleptic traits to differentiate and name their varieties [9, 39]. Remarkably, the number of names recorded in our study was relatively small, with only 31 local names for 132 accessions. Etymologically, only six out of 31 appear to be directly related to names of specific varieties. The main trait used to identify and or name varieties was the color of the skin of the tuber and the color of the vine. Indeed, farmers use phenotype to distinguish the plant [16]. In our study, the farmers were sometimes unable to identify the sweet potato varieties from above ground plant parts alone (vine or leaves) without the root tuber. Similar observations were made by Shigeta [40] for sweet potato landraces in Ethiopia, and by Elias *et al*. [41], for cassava varieties among Makushi Ameridins in Guyana. But, most of the names used for sweet potato varieties clearly identify the species in different languages. Sweet potato is not a major crop in Togo or Senegal, and some farmers may thus not pay much attention to the phenotypic variability of the plant.

### Genetic clusters and morphologic traits

In this study, we demonstrated that the morphological traits of sweet potato were associated with genetic groups. The most significantly associated traits are qualitative like color of the skin root and quantitative like number of leaves. Consequently, a different set of traits found in each genetic group may reflect random variation of phenotype or selection by farmers. At this stage, we could not clearly differentiated these two hypotheses. The importance of these phenotype traits for breeders is mentioned in many studies [16, 42]. Identifying specific morphological traits related to the genetic groups is a very useful way to study diversity in the field, and morphology could help design conservation strategies that maximize genetic diversity.

## Conclusion

The genetic diversity of West African sweet potato is structured by geography, climate, genetics and morphology. This original structure (genetic, morphologic and climate) allows us to suggest the hypothesis that a genetic group associated today with a specific set of climate variable, might performed well in geographic area showing projected future climate with similar characteristics. This hypothesis of specific climatic adaptation could be tested and if validated suggest possible strategy of crop adaptation to future conditions. The high diversity observed in West Africa might suggests that sweet potato was also introduced in the region rather than simply being the result of diffusion after its introduction in East Africa. This suggest another interesting hypothesis to be tested about the diffusion of sweet potato in Africa. More thorough sampling throughout Africa is now needed to assess such hypotheses.

## Supporting information

S1 Fig Method used to choose the number of PCA axes. The number of PCA chosen could be based on (a) a score function which indicated the appropriate number of PCA axes to be retained. In our case, the graph proposed 3 axes (PCs); or (b) the percentage of variance explained; 74.7% of variance was chosen which corresponded to 12 axes (PCs).
(TIFF)

S2 Fig The choice of the number of groups. The graph shows the Bayesian information criterion (BIC) for increasing values of K (1 to 20) which corresponds to the number of clusters to choose. The lowest value of the graph was not clear. To choose K, which describes our dataset well, we ran successive Ks (3, 4, 5, 6) to run DAPC. Finally K = 5 was chosen because it was the best assignment for each sample.
(TIFF)

S3 Fig DAPC representation of successive Ks. The method we used to choose the appropriate value of K, which explained our dataset well. The group subdivision obtained was clear when we moved from K = 3 to K = 4 and K = 5. But when K = 6 the groups were not clearly delimited in the two datasets. The two datasets showed approximately the same thing.
(TIFF)

S4 Fig Number of group assumed by STRUCTURE and delta K (ΔK) value. The result shows that the maximum ΔK value is found with K = 5. This result confirms that K = 5 is the best and appropriate number of groups to be returned.
(PDF)

S5 Fig Accessions linked based on DAPC analysis with SSR data. The diagram shows probable membership of the five groups (K1, K2, K3, K4 and K5) determined by the DAPC analysis. An individual is represented as a vertical bar. The colors correspond to the five groups (red: group one, yellow: group two, green: group three, blue: group four, and pink: group five).
(TIFF)

S6 Fig Principal component analysis to distinguish the villages according to climatic conditions based on BIOCLIM data values. The sampled village arearranged following the first axis according to a climatic gradient from the humid south (DANYI PEYEYEME) to the arid tropical north (GABONBONG) of Togo, followed by the Saharan climat of Senegal (DAGANA).
(TIF)

S1 Table Passport data of the sweet potato samples used on the study. Field code, extraction code, genetic group, local name, village, latitude, longitude and country are shown.
(PDF)

S2 Table PCR conditions, number of alleles per locus on the two data analyze. The table reports the number of alleles per locus as recorded according the two data analyze with GeneMapper software. Slight difference was observed on the total number of alleles: 90 for the first dataset and 83 for the second. But the similar result was funded for all test running with the two dataset.
(PDF)

S3 Table Number of alleles in 132 samples by SSRs locus in different countries. America, Oceania and West Africa with the two datasets. The table shown the SSRs markers used, the numbers of alleles of each markers, the total number of allele found on each continent and their means. (i) Initial dataset (s) second dataset (1) represents the number of alleles on the 132 samples chosen randomly on data base of Roullier *et al* (2011).
(PDF)

S4 Table Diversity among countries. The table show the p-value obtained with Wilcoxon-Paired test between different dataset. The difference is significant when p-value < 0.05.
(PDF)

S5 Table Comparison of DAPC and STRUCTURE result for k = 5. The table reports the membership probability according to structure analyse of each individual and genetic group appurtenance as defined by DAPC results.
(PDF)

S6 Table Kruskall-Wallis test between genetic group and agro-morphological traits. A total of 21 agro-morphological traits were used. The two datasets (initial dataset and second dataset) were used. The value corresponds to p-value of each traits. In grey significant traits p-value < 0.0024 (Bonferroni corrected p-value). The k-mean cluster, K from 3 to 5 for each dataset was tested. The total of significant traits of each model for the two datasets was mentionned.
(PDF)

S7 Table Individuals found in the same group when the two datasets were run. This table shows that the two datasets produce approximately the same results: 92% of the samples are allocated to same group in the two datasets. The color or the letter for the same column of the same K for the two datasets was not identical, this show that the samples have changed cluster. Using this method, only 12 individuals have changed their place for K = 3, 6 for K = 4 and 16 for K = 5.
(PDF)

S8 Table Wilcoxon-paired test between the genetic groups. A p-value < 5% was considered to be significant. The trait is specific to one group when the p-value is significant in each paired comparison with the same trait.
(PDF)
