# Supplementary figures and images for: Structure of sweet potato (Ipomoea batatas) diversity in West Africa covaries with a climatic gradient
Source: PLoS One. 2017 May 26;12(5):e0177697. doi: 10.1371/journal.pone.0177697 (PMC5446114; doi:10.1371/journal.pone.0177697)

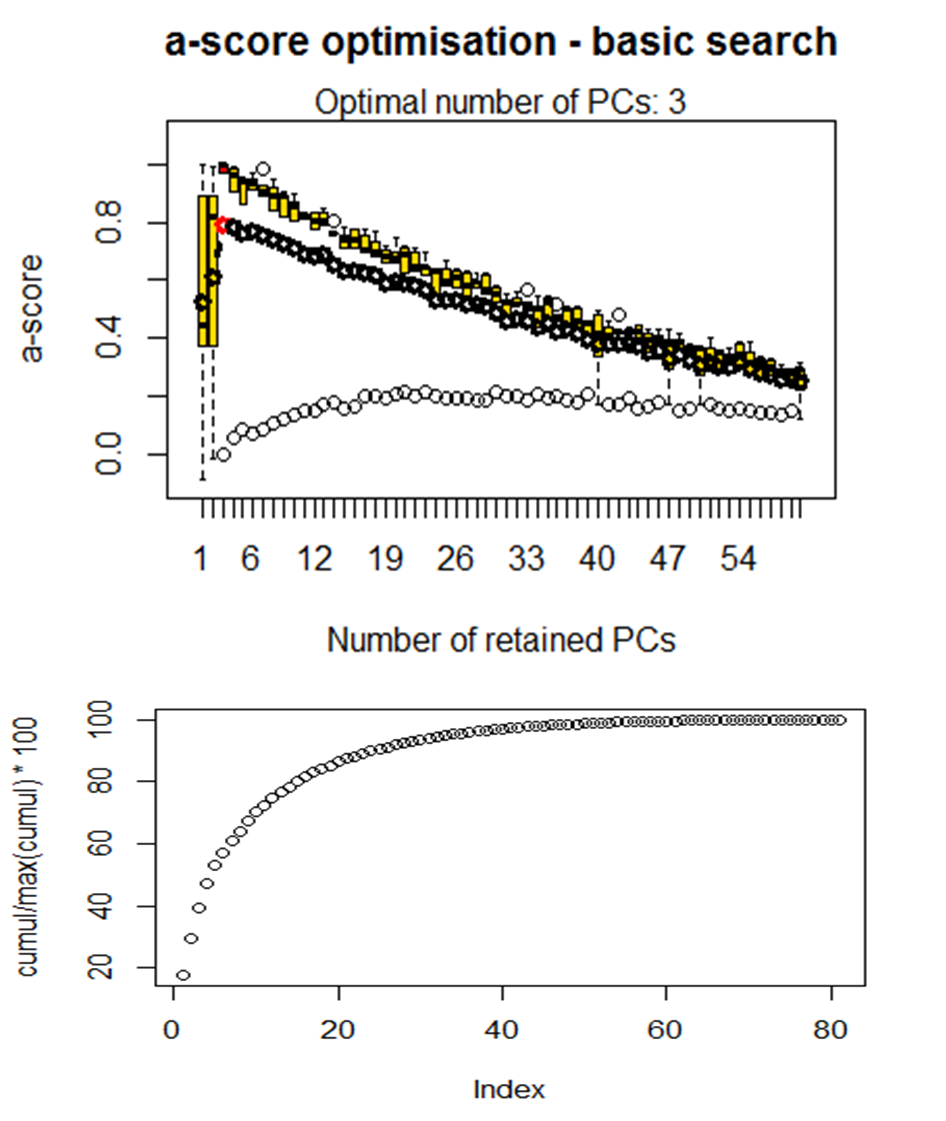

Supplement: S1 Fig — The number of PCA chosen could be based on (a) a score function which indicated the appropriate number of PCA axes to be retained. In our case, the graph proposed 3 axes (PCs); or (b) the percentage of variance explained; 74.7% of variance was chosen which corresponded to 12 axes (PCs). (TIFF) [file pone.0177697.s001.tiff]

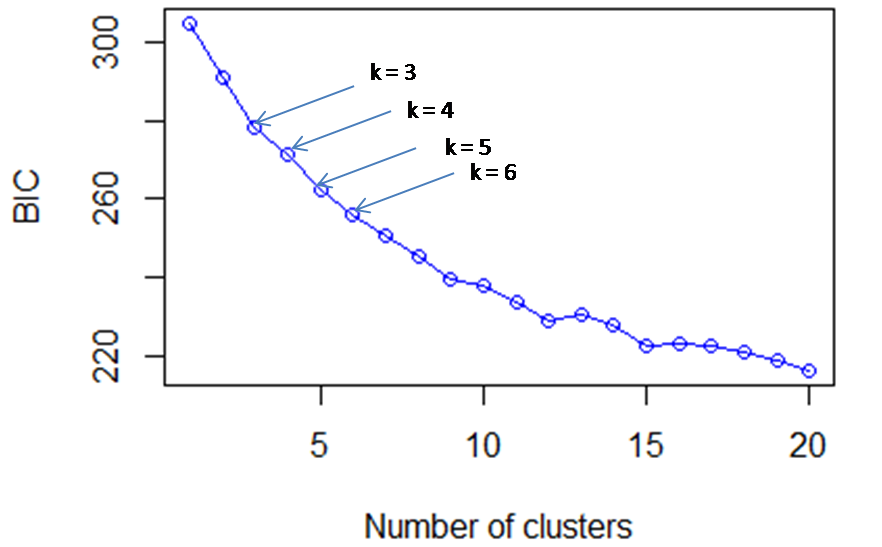

Supplement: S2 Fig — The graph shows the Bayesian information criterion (BIC) for increasing values of K (1 to 20) which corresponds to the number of clusters to choose. The lowest value of the graph was not clear. To choose K, which describes our dataset well, we ran successive Ks (3, 4, 5, 6) to run DAPC. Finally K = 5 was chosen because it was the best assignment for each sample. (TIFF) [file pone.0177697.s002.tiff]

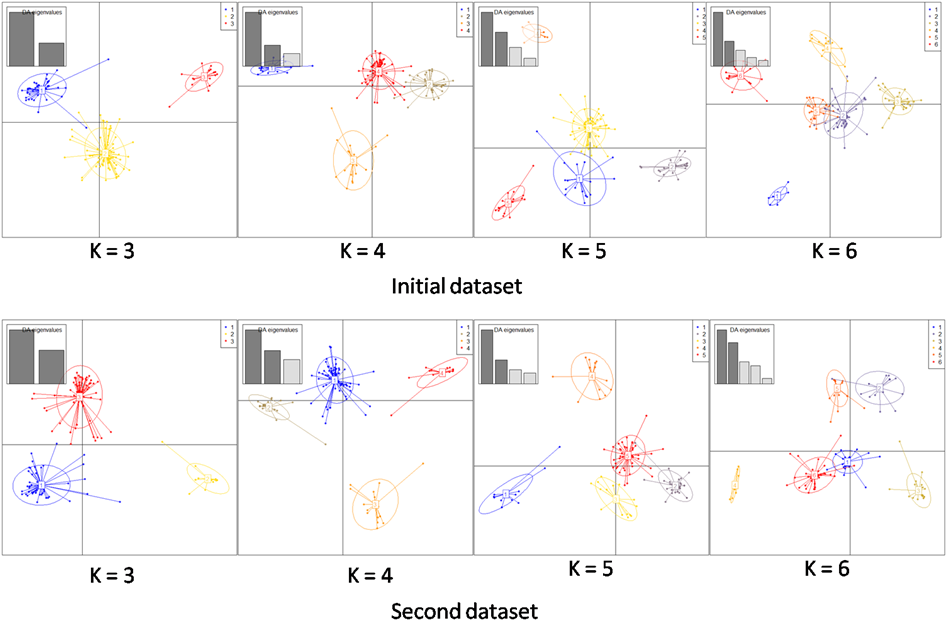

Supplement: S3 Fig — The method we used to choose the appropriate value of K, which explained our dataset well. The group subdivision obtained was clear when we moved from K = 3 to K = 4 and K = 5. But when K = 6 the groups were not clearly delimited in the two datasets. The two datasets showed approximately the same thing. (TIFF) [file pone.0177697.s003.tiff]

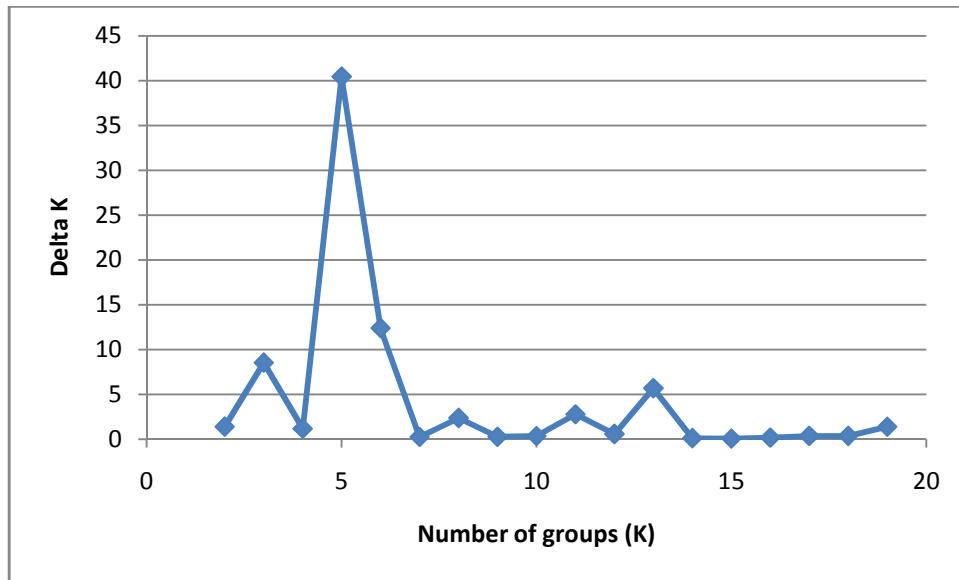

Supplement: S4 Fig — The result shows that the maximum ΔK value is found with K = 5. This result confirms that K = 5 is the best and appropriate number of groups to be returned. (PDF) [file pone.0177697.s004.pdf]

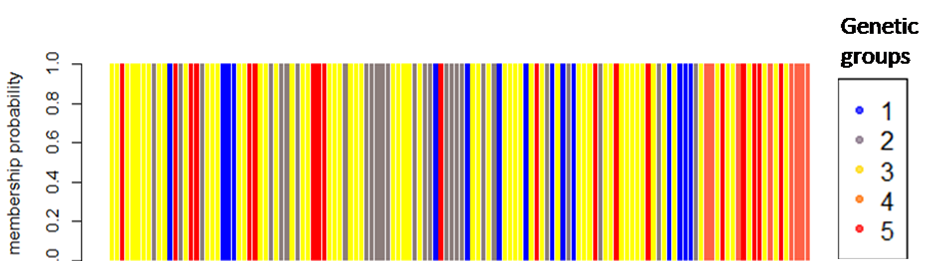

Supplement: S5 Fig — The diagram shows probable membership of the five groups (K1, K2, K3, K4 and K5) determined by the DAPC analysis. An individual is represented as a vertical bar. The colors correspond to the five groups (red: group one, yellow: group two, green: group three, blue: group four, and pink: group five). (TIFF) [file pone.0177697.s005.tiff]

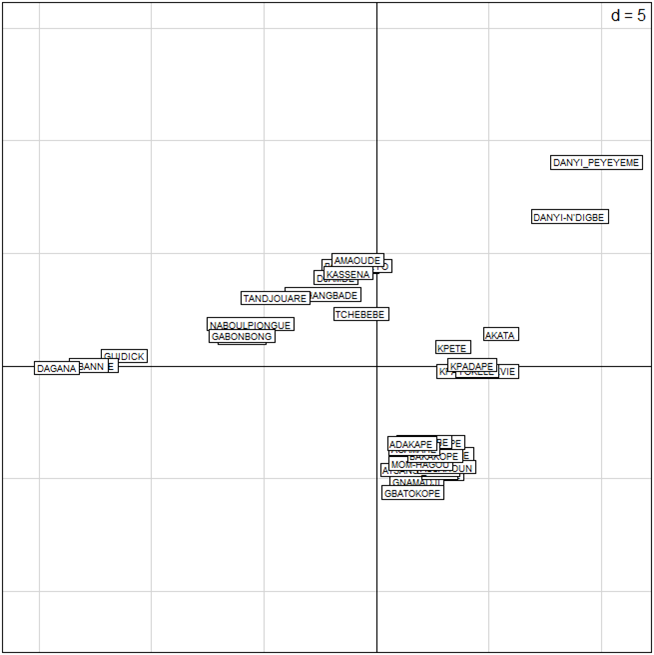

Supplement: S6 Fig — The sampled village arearranged following the first axis according to a climatic gradient from the humid south (DANYI PEYEYEME) to the arid tropical north (GABONBONG) of Togo, followed by the Saharan climat of Senegal (DAGANA). (TIF) [file pone.0177697.s006.tif]
