# Supplementary material for: Structure of sweet potato (Ipomoea batatas) diversity in West Africa covaries with a climatic gradient
Source: PLoS One. 2017 May 26;12(5):e0177697. doi: 10.1371/journal.pone.0177697 (PMC5446114; doi:10.1371/journal.pone.0177697)
Supplement: S1 Table — Field code, extraction code, genetic group, local name, village, latitude, longitude and country are shown. (PDF) [file pone.0177697.s007.pdf]

S1 Table. Passport data of the sweet potato samples used on the study

Field code	Extration code	Local name	Village	Latitude	Longitude	Country
GAB	1	Dankadou	GABONBONG	N11°01'22.1"	E000°04'56.7"	TOGO
GAB1	2	Dankadou	GABONBONG	N11°01'22.1"	E000°04'56.7"	TOGO
GAB2	3	Dankadou	GABONBONG	N11°01'22.1"	E000°04'56.7"	TOGO
GAB3	4	Dankadou	GABONBONG	N11°01'22.1"	E000°04'56.7"	TOGO
NAB	5	Dankalè	NABOULPIONGUE	N10°56'20.8"	E000°08'01.3"	TOGO
NAB2	6	Dankalè	NABOULPIONGUE	N10°56'20.8"	E000°08'01.3"	TOGO
NAB3	7	Dankalè	NABOULPIONGUE	N10°56'20.8"	E000°08'01.3"	TOGO
NAK	8	Kourkou	NAKI-EST	N10°43'31.3"	E000°22'37.6"	TOGO
NAK1	9	Kourkou	NAKI-EST	N10°43'31.3"	E000°22'37.6"	TOGO
NAK3	10	Kourkou	NAKI-EST	N10°43'31.3"	E000°22'37.6"	TOGO
TAN	11	Dinkedout	TANDJOURE	N10°40'15.4"	E000°12'19.6"	TOGO
TAN1	12	Dinkedouk	TANDJOUARE	N10°40'15.4"	E000°12'19.6"	TOGO
TAN2	13	Dinkedout	TANDJOUARE	N10°40'15.4"	E000°12'19.6"	TOGO
DJA	14	Awouhè	DJAMDE	N09°30'50.6"	E001°02'40.0"	TOGO
DJA2	15	Awouhè	DJAMDE	N09°30'50.6"	E001°02'40.0"	TOGO
DJA3	16	Awouhè	DJAMDE	N09°30'50.6"	E001°02'40.0"	TOGO
DJA4	17	Awouhè	DJAMDE	N09°30'50.6"	E001°02'40.0"	TOGO
DJA5	18	Tomba	DJAMDE	N09°30'50.6"	E001°02'40.0"	TOGO
ATC1	19	Tomba	ATCHANGBADE	N09°28'39.4"	E001°08'17.2"	TOGO
ATC2	20	Tomba	ATCHANGBADE	N09°28'39.4"	E001°08'17.2"	TOGO
BOTCH1	21	Atonini	BOTCHOLEYO	N08°56'02.0"	E001°05'48.8"	TOGO
TCHAL1	23	Atononi	TCHALO	N08°55'43.9"	E001°07'03.9"	TOGO
TCHAL2	24	Atonini	TCHALO	N08°55'43.9"	E001°07'03.9"	TOGO
TCHAL3	25	Atonini	TCHALO	N08°55'43.9"	E001°07'03.9"	TOGO
TCHAL4	26	Atonini	TCHALO	N08°55'43.9"	E001°07'03.9"	TOGO

TCHAL5	27	Atonini	TCHALO	N08°55'43.9"	E001°07'03.9"	TOGO
TCHAL6	28	Atonini	TCHALO	N08°55'43.9"	E001°07'03.9"	TOGO
TCHAL7	29	Atonini	TCHALO	N08°55'43.9"	E001°07'03.9"	TOGO
TCH1	30	Awihè	TCHEBEBE	N08°26'16.0"	E000°59'30.4"	TOGO
TCH2	31	Awihè	TCHEBEBE	N08°26'16.0"	E000°59'30.4"	TOGO
TCH3	32	Awihè	TCHEBEBE	N08°26'16.0"	E000°59'30.4"	TOGO
TCH4	33	Awihè	TCHEBEBE	N08°26'16.0"	E000°59'30.4"	TOGO
TCH5	34	Awihè	TCHEBEBE	N08°26'16.0"	E000°59'30.4"	TOGO
KPA-T1	35	Djété	KPALIME TSEVIE	N06°55'22.4"	E000°38'31.0"	TOGO
KPA-T2	36	Djété	KPALIME TSEVIE	N06°55'22.4"	E000°38'31.0"	TOGO
KPA-T3	37	Djété	KPALIME TSEVIE	N06°55'22.4"	E000°38'31.0"	TOGO
KPA-T4	38	Djété	KPALIME TSEVIE	N06°55'22.4"	E000°38'31.0"	TOGO
KPA-T5	39	Djété	KPALIME TSEVIE	N06°55'22.4"	E000°38'31.0"	TOGO
YOK	40	Nagoté	YOKELE	N06°56'42.1"	E000°39'45.5"	TOGO
YOK1	41	Nagoté	YOKELE	N06°56'42.1"	E000°39'45.5"	TOGO
YOK2	42	Agouté	YOKELE	N06°56'42.1"	E000°39'45.5"	TOGO
YOK3	43	Agouté	YOKELE	N06°56'42.1"	E000°39'45.5"	TOGO
DAN-PE1	44	Anagoté	DANYI PEYEYEME	N07°12'42.1"	E000°41'56.2"	TOGO
DAN-PE2	45	Anago	DANYI PEYEYEME	N07°12'42.1"	E000°41'56.2"	TOGO
DAN-PE3	46	Anago	DANYI PEYEYEME	N07°12'42.1"	E000°41'56.2"	TOGO
DAN-PE4	47	Djété	DANYI PEYEYEME	N07°12'42.1"	E000°41'56.2"	TOGO
AKA	48	Nagoté	AKATA	N07°02'14.6"	E000°42'20.5"	TOGO
AKA1	49	Nagoté	AKATA	N07°02'14.6"	E000°42'20.5"	TOGO
AKA2	50	Nago	AKATA	N07°02'14.6"	E000°42'20.5"	TOGO
AKA3	51	Tombolo	AKATA	N07°02'14.6"	E000°42'20.5"	TOGO
AKA4	52	Djété	AKATA	N07°02'14.6"	E000°42'20.5"	TOGO
KPE-TS	53	Djété	KPELE-TSIKO	N07°07'37.6"	E000°42'13.3"	TOGO
KPE-TS1	54	Djété	KPELE-TSIKO	N07°07'37.6"	E000°42'13.3"	TOGO
KPE-TS2	55	Djété	KPELE-TSIKO	N07°07'37.6"	E000°42'13.3"	TOGO

ASS1	56	Tombolo	ASSAHOUN	N06°27'56.0"	E000°54'16.1"	TOGO
ASS2	57	Tombolo	ASSAHOUN	N06°27'56.0"	E000°54'16.1"	TOGO
BAK1	58	Tombolo	BAKAKOPE	N06°39'43.5"	E000°54'20.5"	TOGO
AGBOD1	59	Anago	AGBODJEKPOE	N 06°39'57.8"	E001° 09'44.7"	TOGO
AGBOD2	60	Nago Kadjin	AGBODJEKPOE	N 06°39'57.8"	E001° 09'44.7"	TOGO
AGBOD3	61	Anago	AGBODJEKPOE	N 06°39'57.8"	E001° 09'44.7"	TOGO
AGBOD4	62	Djété	AGBODJEKPOE	N 06°39'57.8"	E001° 09'44.7"	TOGO
AGBOD5	63	Djété	AGBODJEKPOE	N 06°39'57.8"	E001° 09'44.7"	TOGO
AGBOD6	64	Djété	AGBODJEKPOE	N 06°39'57.8"	E001° 09'44.7"	TOGO
AGBOD7	65	Djété	AGBODJEKPOE	N 06°39'57.8"	E001° 09'44.7"	TOGO
GNA	66	Agbozoumeka	GNAMADJI	N06° 15'24.4"	E001°19'05.3"	TOGO
GNA1	67	Agbozoumeka	GNAMADI	N06° 15'24.4"	E001°19'05.3"	TOGO
GNA2	68	Agbozoumeka	GNAMADJI	N06° 15'24.4"	E001°19'05.3"	TOGO
GNA3	69	Agbozoumeka	GNAMADJI	N06° 15'24.4"	E001°19'05.3"	TOGO
GNA4	70	Kafan	GNAMADJI	N06° 15'24.4"	E001°19'05.3"	TOGO
GNA5	71	Kafan	GNAMADJI	N06° 15'24.4"	E001°19'05.3"	TOGO
GNA6	72	Kafan	GNAMADJI	N06° 15'24.4"	E001°19'05.3"	TOGO
GNA7	73	Kafan	GNAMADJI	N06° 15'24.4"	E001°19'05.3"	TOGO
GNA8	74	Kpédévika	GNAMADJI	N06° 15'24.4"	E001°19'05.3"	TOGO
GNA9	75	Kpédévika	GNAMADJI	N06° 15'24.4"	E001°19'05.3"	TOGO
GNA10	76	Kpédévika	GNAMADJI	N06° 15'24.4"	E001°19'05.3"	TOGO
GNA11	77	Djété	GNAMADJI	N06° 15'24.4"	E001°19'05.3"	TOGO
GNA12	78	Djété	GNAMADJI	N06° 15'24.4"	E001°19'05.3"	TOGO
GNA13	79	Djété	GNAMADJI	N06° 15'24.4"	E001°19'05.3"	TOGO
GNA14	80	Djété	GNAMADJI	N06° 15'24.4"	E001°19'05.3"	TOGO
GNI1	81	Tombolo	GNIGBE	N06°42'06.6"	E001°08'14.3"	TOGO
GNI2	82	Tombolo	GNIGBE	N06°42'06.6"	E001°08'14.3"	TOGO
GNI3	83	Djété	GNIGBE	N06°42'06.6"	E001°08'14.3"	TOGO
GNI4	84	Djété	GNIGBE	N06°42'06.6"	E001°08'14.3"	TOGO

GNI5	85	Djété	GNIGBE	N06°42'06.6"	E001°08'14.3"	TOGO
GNI6	86	Djété	GNIGBE	N06°42'06.6"	E001°08'14.3"	TOGO
GNI7	87	Djété	GNIGBE	N06°42'06.6"	E001°08'14.3"	TOGO
GNI8	88	Djété	GNIGBE	N06°42'06.6"	E001°08'14.3"	TOGO
GNI9	89	Djété	GNIGBE	N06°42'06.6"	E001°08'14.3"	TOGO
GNI10	90	Djété	GNIGBE	N06°42'06.6"	E001°08'14.3"	TOGO
MOM-HA	91	Nago	MOM-HAGOU	N06°29'30.8"	E001°33'30.9"	TOGO
KPE-BE3	92	Djété	KAGNIKPEDJI	N06°38'48.4"	E001°10'22.4"	TOGO
GBA1	93	Djété	GBATOKOPE	N06°14'31.6"	E001° 32'05.5"	TOGO
DANN1	94	Djété	DANYI-N'DIGBE	N07°08'30.1"	E000°40'35.5"	TOGO
DANN2	95	Djété	DANYI-N'DIGBE	N07°08'30.1"	E000°40'35.5"	TOGO
ATS-DZ1	97	Djété	ATSANSI DEDZI	N06°27'26.9"	E001°32'50.9"	TOGO
ATS-DZ2	98	Djété	ATSANSI DEDZI	N06°27'26.9"	E001°32'50.9"	TOGO
ATS-DZ3	99	Djété	ATSANSI DEDZI	N06°27'26.9"	E001°32'50.9"	TOGO
BAD1	100	Djété	BADJA	N06°23'81.8"	E000°59'67.6"	TOGO
BAD2	101	Djété	BADJA	N06°23'81.8"	E000°59'67.6"	TOGO
GBAV	102	Djété	GBAVE	N 06°20'08.0"	E001°01'40.0"	TOGO
ADRA1	103	Djété	ADRALAKOPE	N06°41'22.0"	E001°08'59.1"	TOGO
ADRA2	104	Djété	ADRALAKOPE	N06°41'22.0"	E001°08'59.1"	TOGO
AMA3	106	Djété	AMAOUDE	N09°08'44.2"	E001°09'24.0"	TOGO
KPE2	109	Djété	KPETE	N07°25'05.6"	E000°52'57.6"	TOGO
AMA2	110	Djété	AMAOUDE	N09°08'44.2"	E001°09'24.0"	TOGO
KASS	111	Djété	KASSENA	N08°52'60"	E001°04'60"	TOGO
ADA2	113	Djété	ADAKAPE	N06°49'04.1"	E001°10'52.5"	TOGO
KPA-DA1	114	Djété	KPADAPE	N06°51'0"	E000°36'0"	TOGO
KPE1	115	Djété	KPETE	N07°25'05.6"	E000°52'57.6"	TOGO
AGA2	117	Djété	AGAMAHE	N06°42'40.5"	E001°10'19.1"	TOGO
AGA	118	Djété	AGAMAHE	N06°42'40.5"	E001°10'19.1"	TOGO
GNI11	120	Djété	GNIGBE	N06°42'06.6"	E001°08'14.3"	TOGO

MBA1	SP1	Limalé Diop	MBANN	N16°17'02.2"	WO15°47'02.9"	SENEGAL
MBA2	SP2	Patass bouweekh	MBANN	N16°17'02.2"	WO15°47'02.9"	SENEGAL
GUI1	SP3	Limalé Diop	GUIDICK	N16°07'16.4"	WO15°53'52.9"	SENEGAL
GUI2	SP4	25/44	GUIDICK	N16°07'16.4"	WO15°53'52.9"	SENEGAL
GUI3	SP5	Orange	GUIDICK	N16°07'16.4"	WO15°53'52.9"	SENEGAL
DAGA1	SP6	Patass bouweekh	DAGANA	N16°32'27.1"	WO15°30'35.6"	SENEGAL
DAGA2	SP7	Ganjul	DAGANA	N16°32'27.1"	WO15°30'35.6"	SENEGAL
DAGA3	SP8	Ganjul	DAGANA	N16°32'27.1"	WO15°30'35.6"	SENEGAL
DAGA4	SP9	Ganjul	DAGANA	N16°32'27.1"	WO15°30'35.6"	SENEGAL
SANE1	SP10	Patass bouweekh	SANEITE	N16°14'06"	WO15°47'30.2"	SENEGAL
SANE2	SP11	Abou thiam	SANEITE	N16°14'06"	WO15°47'30.2"	SENEGAL
SANE3	SP12	25/44 ou Ibrahim diop	SANEITE	N16°14'06"	WO15°47'30.2"	SENEGAL
SANE4	SP13	83/176 Tis ou Limalé Diop	SANEITE	N16°14'06"	WO15°47'30.2"	SENEGAL
SANE5	SP14	Thiate	SANEITE	N16°14'06"	WO15°47'30.2"	SENEGAL
SANE6	SP15	Walo ou Faneyi	SANEITE	N16°14'06"	WO15°47'30.2"	SENEGAL
SANE7	SP16	Orange	SANEITE	N16°14'06"	WO15°47'30.2"	SENEGAL
SANE8	SP17	Mlick Diop	SANEITE	N16°14'06"	WO15°47'30.2"	SENEGAL
SANE9	SP18	Patass bouweekh	SANEITE	N16°14'06"	WO15°47'30.2"	SENEGAL
SANE10	SP19	Patass bouweekh	SANEITE	N16°14'06"	WO15°47'30.2"	SENEGAL
SANE11	SP20	Patass bouweekh	SANEITE	N16°14'06"	WO15°47'30.2"	SENEGAL
