# Supplementary material for: Structure of sweet potato (Ipomoea batatas) diversity in West Africa covaries with a climatic gradient
Source: PLoS One. 2017 May 26;12(5):e0177697. doi: 10.1371/journal.pone.0177697 (PMC5446114; doi:10.1371/journal.pone.0177697)
Supplement: S2 Table — The table reports the number of alleles per locus as recorded according the two data analyze with GeneMapper software. Slight difference was observed on the total number of alleles: 90 for the first dataset and 83 for the second. But the similar result was funded for all test running with the two dataset. (PDF) [file pone.0177697.s008.pdf]

S2 Table. SSRs markers and PCR conditions

Locus Name	T(°c)	Dye	Run	dilution
J206A	58	NED	A	1/100
J1809E	60	VIC	A	1/100
J544b	60	NED	A	1/100
IbS11	60	FAM	A	1/100
IbR16	60	NED	B	1/100
IbC5	60	NED	B	1/100
Ib297	58	FAM	B	1/50
J522A	58	VIC	B	1/100
J263	58	FAM	C	1/100
J315E	58	NED	C	1/100
J10A	58	VIC	C	1/50
J116a	58	PET	C	1/50
