# Supplementary material for: Structure of sweet potato (Ipomoea batatas) diversity in West Africa covaries with a climatic gradient
Source: PLoS One. 2017 May 26;12(5):e0177697. doi: 10.1371/journal.pone.0177697 (PMC5446114; doi:10.1371/journal.pone.0177697)
Supplement: S3 Table — America, Oceania and West Africa with the two datasets. The table shown the SSRs markers used, the numbers of alleles of each markers, the total number of allele found on each continent and their means. (i) Initial dataset (s) second dataset (1) represents the number of alleles on the 132 samples chosen randomly on data base of Roullier et al (2011). (PDF) [file pone.0177697.s009.pdf]

S3 Table. Number of alleles per locus on the two datasets

	Number of alleles	
locus	Initial dataset	Second dataset
Ibc5	11	12
Ib297	12	10
IbR16	5	5
J1809E	5	5
J206A	7	5
J263	4	4
J522A	7	4
Ibs11	9	10
J544B	5	5
J315E	4	4
J116a	12	11
J10a	9	8
Total	90	83
Mean	7.5	6.91
