# Supplementary material for: Structure of sweet potato (Ipomoea batatas) diversity in West Africa covaries with a climatic gradient
Source: PLoS One. 2017 May 26;12(5):e0177697. doi: 10.1371/journal.pone.0177697 (PMC5446114; doi:10.1371/journal.pone.0177697)
Supplement: S4 Table — The table show the p-value obtained with Wilcoxon-Paired test between different dataset. The difference is significant when p-value < 0.05. (PDF) [file pone.0177697.s010.pdf]

S4 Table. Number of alleles in 132 samples by SSRs locus in different countries with the two datasets

Number of alleles				
locus	West Africa(i)	West Africa(s)	Tropical America (1)	Oceania (1)
Ibc5	11	12	14	9
ib297	12	10	22	17
ibR16	5	5	9	10
J1809E	5	5	9	6
J206A	7	5	7	5
J263	4	4	7	4
J522A	7	4	7	8
ibs11	9	10	12	10
J544B	5	5	8	11
J315E	4	4	9	6
J116a	12	11	16	14
Total	83	75	120	100
Mean	7.36	6.81	10.9	9.09
