# Supplementary material for: Structure of sweet potato (Ipomoea batatas) diversity in West Africa covaries with a climatic gradient
Source: PLoS One. 2017 May 26;12(5):e0177697. doi: 10.1371/journal.pone.0177697 (PMC5446114; doi:10.1371/journal.pone.0177697)
Supplement: S5 Table — The table reports the membership probability according to structure analyse of each individual and genetic group appurtenance as defined by DAPC results. (PDF) [file pone.0177697.s011.pdf]

S5 Table. Diversity among countries

	West Africa (i)	West Africa (s)	Oceania	America
Africa (i)	-			
Africa (s)	0.202	-		
Oceania	0.099	0.049	-	
America	0.008	0.003	0.048	-
