# Supplementary material for: Structure of sweet potato (Ipomoea batatas) diversity in West Africa covaries with a climatic gradient
Source: PLoS One. 2017 May 26;12(5):e0177697. doi: 10.1371/journal.pone.0177697 (PMC5446114; doi:10.1371/journal.pone.0177697)
Supplement: S6 Table — A total of 21 agro-morphological traits were used. The two datasets (initial dataset and second dataset) were used. The value corresponds to p-value of each traits. In grey significant traits p-value < 0.0024 (Bonferroni corrected p-value). The k-mean cluster, K from 3 to 5 for each dataset was tested. The total of significant traits of each model for the two datasets was mentionned. (PDF) [file pone.0177697.s012.pdf]

S6 Table. Comparison of DAPC and STRUCTURE results for K=5

Name	Structure ancestry	Structure ancestry	Structure ancestry	Structure ancestry
T11	0.99	0.00	0.00	0.00
T35	0.99	0.00	0.00	0.00
T36	0.99	0.00	0.00	0.00
T45	0.97	0.01	0.01	0.00
T46	0.98	0.00	0.00	0.00
T47	0.99	0.00	0.00	0.00
T48	0.99	0.00	0.00	0.00
T59	0.99	0.00	0.00	0.00
T61	0.41	0.17	0.25	0.04
T84	0.99	0.00	0.01	0.00
T85	0.43	0.29	0.13	0.01
T109	0.00	0.99	0.00	0.00
T114	0.00	0.94	0.01	0.00
T12	0.01	0.95	0.02	0.01
T24	0.00	0.99	0.00	0.00
T27	0.00	0.99	0.00	0.00
T29	0.01	0.98	0.01	0.01
T37	0.00	0.99	0.00	0.00
T40	0.00	0.99	0.00	0.00
T41	0.00	0.94	0.00	0.02
T42	0.00	0.99	0.00	0.00
T43	0.00	0.99	0.00	0.00
T44	0.02	0.96	0.00	0.01
T49	0.01	0.99	0.00	0.00
T50	0.01	0.97	0.00	0.02
T51	0.01	0.98	0.00	0.01
T54	0.08	0.85	0.02	0.05
T55	0.00	0.99	0.00	0.00
T56	0.01	0.98	0.01	0.01
T57	0.01	0.97	0.01	0.01
T60	0.03	0.91	0.01	0.04
T62	0.00	0.99	0.00	0.00
T71	0.01	0.95	0.01	0.02
T75	0.00	0.99	0.00	0.00
T80	0.00	0.99	0.00	0.00
T90	0.00	0.98	0.01	0.01
T97	0.01	0.37	0.44	0.16
T98	0.01	0.94	0.03	0.01
SP12	0.00	0.00	0.99	0.00
T100	0.00	0.00	0.99	0.00
T113	0.00	0.00	0.99	0.00
T117	0.12	0.01	0.83	0.01
T118	0.01	0.01	0.98	0.00
T2	0.00	0.01	0.97	0.01
T20	0.04	0.02	0.93	0.00
T26	0.01	0.97	0.01	0.01
T31	0.00	0.00	0.99	0.00

T32	0.00	0.00	0.99	0.00
T33	0.03	0.01	0.90	0.00
T53	0.00	0.00	0.99	0.00
T7	0.00	0.00	0.99	0.00
T8	0.05	0.01	0.93	0.01
T83	0.00	0.00	0.99	0.00
T89	0.00	0.00	0.99	0.00
SP16	0.01	0.01	0.93	0.01
SP18	0.00	0.00	0.99	0.00
SP19	0.00	0.00	0.99	0.00
SP4	0.00	0.00	0.99	0.00
T111	0.04	0.02	0.01	0.85
T15	0.00	0.00	0.00	0.99
T16	0.00	0.00	0.00	0.99
T17	0.00	0.00	0.00	0.99
T52	0.00	0.00	0.00	0.99
T58	0.00	0.00	0.00	0.99
T63	0.01	0.00	0.02	0.58
T68	0.00	0.01	0.02	0.66
T72	0.01	0.02	0.01	0.88
T74	0.00	0.00	0.00	0.99
T76	0.02	0.12	0.01	0.84
T94	0.03	0.00	0.01	0.95
T1	0.08	0.01	0.01	0.00
T10	0.12	0.09	0.01	0.01
T101	0.03	0.02	0.01	0.00
T102	0.01	0.03	0.00	0.07
T103	0.01	0.04	0.01	0.01
T104	0.16	0.03	0.01	0.01
T106	0.01	0.26	0.01	0.01
T110	0.01	0.00	0.01	0.01
T115	0.00	0.04	0.02	0.12
T120	0.01	0.13	0.04	0.04
T13	0.01	0.01	0.02	0.00
T14	0.01	0.07	0.01	0.00
T18	0.01	0.00	0.01	0.00
T19	0.01	0.01	0.01	0.01
T21	0.01	0.04	0.00	0.01
T23	0.01	0.02	0.00	0.00
T25	0.01	0.02	0.00	0.00
T28	0.01	0.04	0.00	0.00
T3	0.37	0.01	0.00	0.01
T30	0.02	0.00	0.01	0.00
T34	0.01	0.00	0.01	0.00
T38	0.06	0.02	0.00	0.22
T39	0.03	0.02	0.00	0.24
T4	0.04	0.22	0.02	0.12
T5	0.01	0.10	0.00	0.01
T6	0.06	0.01	0.01	0.01
T64	0.01	0.02	0.03	0.02

T65	0.01	0.13	0.09	0.01
T66	0.01	0.01	0.01	0.01
T67	0.01	0.01	0.01	0.00
T69	0.02	0.02	0.01	0.01
T70	0.02	0.01	0.01	0.00
T73	0.01	0.23	0.01	0.01
T77	0.01	0.02	0.05	0.01
T78	0.02	0.04	0.01	0.00
T79	0.01	0.13	0.00	0.01
T81	0.03	0.01	0.01	0.01
T82	0.02	0.03	0.01	0.01
T86	0.01	0.01	0.01	0.00
T87	0.24	0.12	0.24	0.05
T88	0.01	0.05	0.03	0.24
T9	0.04	0.01	0.01	0.01
T91	0.01	0.01	0.01	0.00
T92	0.01	0.01	0.01	0.40
T93	0.03	0.03	0.02	0.00
T95	0.01	0.01	0.01	0.40
T99	0.01	0.00	0.01	0.01
SP1	0.00	0.00	0.01	0.00
SP10	0.00	0.00	0.01	0.00
SP11	0.00	0.01	0.01	0.20
SP13	0.07	0.03	0.04	0.18
SP14	0.01	0.01	0.05	0.01
SP15	0.00	0.00	0.01	0.00
SP17	0.01	0.01	0.01	0.11
SP2	0.00	0.01	0.01	0.17
SP20	0.00	0.00	0.01	0.00
SP3	0.08	0.04	0.03	0.13
SP5	0.01	0.03	0.21	0.04
SP6	0.00	0.00	0.01	0.00
SP7	0.00	0.00	0.02	0.00
SP8	0.01	0.00	0.05	0.01
SP9	0.01	0.00	0.04	0.01

Structure ancestry	DAPC Group
0.00	1
0.00	1
0.00	1
0.01	1
0.01	1
0.00	1
0.00	1
0.00	1
0.13	1
0.00	1
0.14	1
0.00	2
0.05	2
0.00	2
0.00	2
0.00	2
0.00	2
0.00	2
0.00	2
0.03	2
0.00	2
0.00	2
0.01	2
0.00	2
0.01	2
0.01	2
0.01	2
0.00	2
0.00	2
0.00	2
0.01	2
0.00	2
0.01	2
0.00	2
0.00	2
0.01	2
0.02	2
0.01	2
0.00	2
0.00	3
0.00	3
0.03	3
0.01	3
0.01	3
0.01	3
0.00	3
0.00	3

0.00	3
0.06	3
0.00	3
0.00	3
0.00	3
0.00	3
0.00	3
0.05	3
0.00	3
0.00	3
0.00	3
0.09	4
0.00	4
0.00	4
0.00	4
0.00	4
0.00	4
0.39	4
0.32	4
0.08	4
0.00	4
0.02	4
0.01	4
0.90	5
0.77	5
0.95	5
0.88	5
0.93	5
0.81	5
0.72	5
0.97	5
0.82	5
0.79	5
0.96	5
0.91	5
0.97	5
0.97	5
0.94	5
0.97	5
0.97	5
0.95	5
0.61	5
0.96	5
0.98	5
0.69	5
0.71	5
0.61	5
0.88	5
0.92	5
0.92	5

0.76	5
0.97	5
0.98	5
0.95	5
0.96	5
0.75	5
0.91	5
0.92	5
0.85	5
0.95	5
0.93	5
0.98	5
0.35	5
0.67	5
0.93	5
0.97	5
0.58	5
0.92	5
0.58	5
0.97	5
0.99	5
0.99	5
0.79	5
0.67	5
0.93	5
0.99	5
0.87	5
0.81	5
0.99	5
0.73	5
0.72	5
0.99	5
0.97	5
0.94	5
0.94	5
