# Supplementary material for: Structure of sweet potato (Ipomoea batatas) diversity in West Africa covaries with a climatic gradient
Source: PLoS One. 2017 May 26;12(5):e0177697. doi: 10.1371/journal.pone.0177697 (PMC5446114; doi:10.1371/journal.pone.0177697)
Supplement: S7 Table — This table shows that the two datasets produce approximately the same results: 92% of the samples are allocated to same group in the two datasets. The color or the letter for the same column of the same K for the two datasets was not identical, this show that the samples have changed cluster. Using this method, only 12 individuals have changed their place for K = 3, 6 for K = 4 and 16 for K = 5. (PDF) [file pone.0177697.s013.pdf]

Table S7. Individus founded on the same group when runing the two datasets

Initial dataset				Second dataset		
Code	k = 3	k = 4	k = 5	code	k = 3	k = 4
DJA2	a	d	d	DJA2	a	d
DJA3	a	d	d	DJA3	a	d
DJA4	a	d	d	DJA4	a	d
TCHAL2	a	a	a	TCHAL2	a	a
TCHAL4	a	a	a	TCHAL4	a	a
TCHAL5	a	a	a	TCHAL5	a	a
TCHAL7	a	a	a	TCHAL7	a	a
KPA-T3	a	a	a	KPA-T3	a	a
AKA4	a	d	d	AKA4	a	d
BAK1	a	d	d	BAK1	a	d
YOK	a	a	a	YOK	a	a
YOK1	a	a	a	YOK1	a	a
YOK2	a	a	a	YOK2	a	a
YOK3	a	a	a	YOK3	a	a
DAN-PE1	a	a	a	DAN-PE1	a	a
AKA1	a	a	a	AKA1	a	a
AKA2	a	a	a	AKA2	a	a
AKA3	a	a	a	AKA3	a	a
KPE-TS1	a	a	a	KPE-TS1	a	a
KPE-TS2	a	a	a	KPE-TS2	a	a
ASS1	a	a	a	ASS1	a	a
ASS2	a	a	a	ASS2	a	a
AGBOD2	a	a	a	AGBOD2	a	a
AGBOD4	a	a	a	AGBOD4	a	a
GNA2	a	d	d	GNA2	a	d
GNA6	a	d	d	GNA6	a	d
GNA8	a	d	d	GNA8	a	d
GNA10	a	d	d	GNA10	a	d
GNA5	a	a	a	GNA5	a	a
GNA9	a	a	a	GNA9	a	a
GNA14	a	a	a	GNA14	a	a
KPE-BE3	a	d	d	KPE-BE3	a	d
DANN1	a	d	d	DANN1	a	d
DANN2	a	d	d	DANN2	a	d
ATS-DZ1	a	d	d	ATS-DZ1	a	c
KASS	a	d	d	KASS	a	d
TAN1	a	a	a	TAN1	a	a
GNI10	a	a	a	GNI10	a	a
ATS-DZ2	a	a	a	ATS-DZ2	a	a
KPE2	a	a	a	KPE2	a	a
KPA-DA1	a	a	a	KPA-DA1	a	a
GAB3	a	a	b	GAB3	a	b
AGBOD5	b	d	d	AGBOD5	a	d
GAB	b	b	b	GAB	b	b
GAB2	b	b	b	GAB2	b	b
TAN	b	b	b	TAN	b	b
TAN2	b	b	b	TAN2	b	b

DJA	b	b	b	DJA	b	b
DJA5	b	b	b	DJA5	b	b
ATC1	b	b	b	ATC1	b	b
BOTCH1	b	b	b	BOTCH1	b	b
TCHAL1	b	b	b	TCHAL1	b	b
TCHAL3	b	b	b	TCHAL3	b	b
TCHAL6	b	b	b	TCHAL6	b	b
TCH1	b	b	b	TCH1	b	b
TCH5	b	b	b	TCH5	b	b
KPA-T1	b	b	b	KPA-T1	b	b
KPA-T2	b	b	b	KPA-T2	b	b
KPA-T4	b	b	b	KPA-T4	b	b
KPA-T5	b	b	b	KPA-T5	b	b
DAN-PE2	b	b	b	DAN-PE2	b	b
DAN-PE3	b	b	b	DAN-PE3	b	b
DAN-PE4	b	b	b	DAN-PE4	b	b
AKA	b	b	b	AKA	b	b
AGBOD1	b	b	b	AGBOD1	b	b
AGBOD3	b	b	b	AGBOD3	b	b
AGBOD6	b	b	b	AGBOD6	b	b
GNA	b	b	b	GNA	b	b
GNA1	b	b	b	GNA1	b	b
GNA3	b	b	b	GNA3	b	b
GNA4	b	b	b	GNA4	b	b
AGBOD7	b	b	b	AGBOD7	a	b
GNA11	b	b	b	GNA11	b	b
GNA12	b	b	b	GNA12	b	b
GNA13	b	a	b	GNA13	a	b
GNI1	b	b	b	GNI1	b	b
GNI2	b	b	b	GNI2	b	b
GNI4	b	b	b	GNI4	b	b
GNI5	b	b	b	GNI5	b	b
GNI6	b	b	b	GNI6	b	b
GNI7	b	b	b	GNI7	b	b
MOM-HA	b	b	b	MOM-HA	b	b
GBA1	b	b	b	GBA1	b	b
ATS-DZ3	b	b	b	ATS-DZ3	b	b
BAD2	b	b	b	BAD2	b	b
GBAV	b	b	b	GBAV	b	b
ADRA1	b	b	b	ADRA1	b	b
ADRA2	b	b	b	ADRA2	b	b
GNI8	b	b	b	GNI8	a	b
AMA3	b	a	b	AMA3	a	b
AMA2	b	b	b	AMA2	b	b
KPE1	b	b	b	KPE1	b	b
GNA7	b	a	b	GNA7	a	b
GNI11	b	b	b	GNI11	a	b
MBA2	b	d	d	MBA2	a	d
SANE2	b	d	d	SANE2	a	d
SANE8	b	d	d	SANE8	a	d

NAB	b	a	b	NAB	a	b
NAB2	b	b	b	NAB2	b	b
NAK1	b	b	b	NAK1	b	b
NAK3	b	b	b	NAK3	b	b
GUI3	b	b	b	GUI3	a	b
GUI1	b	b	b	GUI1	b	b
SANE4	b	b	b	SANE4	b	b
SANE5	b	b	b	SANE5	b	b
MBA1	b	b	e	MBA1	b	b
DAGA1	b	b	e	DAGA1	b	b
DAGA2	b	b	e	DAGA2	b	b
DAGA3	b	b	e	DAGA3	b	b
DAGA4	b	b	e	DAGA4	b	b
SANE1	b	b	e	SANE1	b	b
SANE6	b	b	e	SANE6	b	b
SANE11	b	b	e	SANE11	b	b
GAB1	c	c	c	GAB1	c	c
NAB3	c	c	c	NAB3	c	c
NAK	c	c	c	NAK	c	c
ATC2	c	c	c	ATC2	c	c
TCH2	c	c	c	TCH2	c	c
TCH3	c	c	c	TCH3	c	c
TCH4	c	c	c	TCH4	c	c
KPE-TS	c	c	c	KPE-TS	c	c
GNI3	c	c	c	GNI3	c	c
GNI9	c	c	c	GNI9	c	c
BAD1	c	c	c	BAD1	c	c
ADA2	c	c	c	ADA2	c	c
AGA2	c	c	c	AGA2	c	c
AGA	c	c	c	AGA	c	c
GUI2	c	c	c	GUI2	c	c
SANE3	c	c	c	SANE3	c	c
SANE7	c	c	c	SANE7	c	c
SANE9	c	c	c	SANE9	c	c
SANE10	c	c	c	SANE10	c	c

k = 5
d
d
d
a
a
a
a
a
d
d
a
a
a
a
a
a
a
a
a
a
a
a
a
a
d
d
d
d
a
a
a
d
d
d
c
d
a
a
a
a
a
b
d
b
b
b
b

b
b
b
b
b
b
b
b
b
b
b
b
b
b
b
b
b
b
b
b
b
b
b
b
e
e
e
a
b
b
b
b
b
b
b
e
b
b
b
b
b
b
a
b
b
a
a
e
e
e

a
b
b
b
e
b
b
e
e
e
e
e
e
e
e
e
c
c
c
c
c
c
c
c
c
c
c
c
c
c
c
c
c
c
c
