# Supplementary material for: Structure of sweet potato (Ipomoea batatas) diversity in West Africa covaries with a climatic gradient
Source: PLoS One. 2017 May 26;12(5):e0177697. doi: 10.1371/journal.pone.0177697 (PMC5446114; doi:10.1371/journal.pone.0177697)
Supplement: S8 Table — A p-value < 5% was considered to be significant. The trait is specific to one group when the p-value is significant in each paired comparison with the same trait. (PDF) [file pone.0177697.s014.pdf]

S8 Table. Wilcoxon Paired test between the genetics groups

	Group 1	Group 2	Group 3
	Leaf vein colour		
Group 2	0.18		
Group 3	0.18	0.873	
Group 5	0.18	0.00058	0.00058
	Skin principal colour		
Group 2	0.679		
Group 3	0.00042	0.00063	
Group 5	0.0019	0.0024	0.67
	Leaf lobe type		
Group 2	0.063		
Group 3	1	0.0027	
Group 5	1	0.05	1
	Leaf outline		
Group 2	0.05		
Group 3	0.98	0.0015	
Group 5	0.032	0.827	0.002
	Leaf lobe number		
Group 2	0.03		
Group 3	1	0.00024	
Group 5	1	0.027	1
	Leaf central lobe type		
Group 2	0.02		
Group 3	1	0.001	
Group 5	1	0.025	1
	Immature leaf colour		
Group 2	0.06		
Group 3	0.55	0.066	
Group 5	0.007	0.017	0.00075
	Pubescence		
Group 2	9,00E-05		
Group 3	0.00052	0.0058	
Group 5	0.08	0.0002	0.033
	Leaf petiole colour		
Group 2	0.68		
Group 3	0.129	0.0039	
Group 5	0.557	0.686	0.0155

The p-value < 5% was considered as significant. The trait is specific to one group when the p-value is significant in each paired comparison with the same trait
